# Supplementary material for: Effect of Ramadan Fasting on Weight and Body Composition in Healthy Non-Athlete Adults: A Systematic Review and Meta-Analysis
Source: Nutrients. 2019 Feb 24;11(2):478. doi: 10.3390/nu11020478 (PMC6412279; doi:10.3390/nu11020478)
Supplement: Supplementary file 1 [file nutrients-11-00478-s001.pdf]

Supplementary materials

Effect of Ramadan fasting on weight and body composition in healthy adult non-athletes: a systematic review and meta-analysis

Hamish A. Fernando[1], Jessica Zibellini[1], Rebecca A. Harris[1], Radhika V. Seimon[1*], Amanda Sainsbury[1*#]

[1]The University of Sydney, The Boden Institute of Obesity, Nutrition, Exercise & Eating Disorders, Faculty of Medicine and Health, Charles Perkins Centre, NSW 2006, Australia

#Equal contribution

***Corresponding author: Amanda Salis (nee Sainsbury)**

Email: amanda.salis@sydney.edu.au

Phone: +61 423 777 801

Table S1a. Publication details and characteristics of participants of all included studies.

Publication	Sample size			Age (years)	Pre-R BMI (kg/m²)	Year of study	Location of study	Fasting duration (hours)	Timing of measurements (relative to the Ramadan fast)		
	M	F	Total						Pre-R	Post-R	Follow-up
Fedail 1982 (28)	20	4	24	21-40			Bristol, UK/Khartoum, Sudan	16	Day 1	Last day	
Husain 1987 [M] (29)	12		21	20-45			Malaysia	14.5	1-7 days before	Just after	
Husain 1987 [F] (29)		9									
Takruri 1989 [Ow] (11)	50	25	75	19-59			Jordan	16.5	1 day before	Day 29	
Takruri 1989 [Nw] (11)	24	14	38								
Azizi 1992 (30)	9		9	23-54		1983	Tehran, Iran	17	7 days before	Day 29	28 days after
Sweileh 1992 (31)	7	1	8	22-35			USA		7 days before	Day 21-28	
Maislos 1993 (32)	16	8	24	18-45			Israel			Day 26, 27	28 days after
El-Ati 1995 (33)		16	16	25-39	22.7±1.2		Tunisia		2 days before	Day 28	28 days after
Adlouni 1998 (34)	32		32	25-50			Casablanca, Morocco		7 days before	Day 29	28 days after
Bilto 1998 (35)	34	9	43	20-48		1997	Jordan	11*	1-7 days before	Day 21-28	
Finch 1998 (36)	15	26	41	19-63		1996	Reading, UK	10.5*	Just before	Just after	28 days after
Kayikcioglu 1998 (37)	32		32	22.3±2.9		1998	Turkey		Beginning of Ramadan	End of Ramadan	
Fakhrzadeh 2003 [M] (38)	50		91	20.8±3.1	21.8±2.6	2000	Tehran, Iran	11.5	7 days before	Day 28	
Fakhrzadeh 2003 [F] (38)		41			24.0±4.5						
Kassab 2003 [N-Ob] (12)	26		44	18-45	22.5±2.0		Bahrain	13	Day 1	Day 28	14 days after
Kassab 2003 [Ob] (12)		18			33.1±0.4						
Kassab 2004 (39)		46	46	18-45	25.3±4.8		Bahrain	13*	Day 1	Day 28	14 days after
Rahman 2004 (40)	20		20	38.3±4.1	24.2±2.5	1998-1999	Bangladesh	12	1 day before	Day 26	28 days after
Saleh 2004 (18)	15	88	103	15-52	39.7±6.4	2002-2003	Egypt	10.5	Before onset	End	28 days after
Yucel 2004 [M] (41)	21		38	20-45	25.2±4.2	2002	Turkey	13	Just before	Just after	
Yucel 2004 [F] (41)		17			24.5±4.7						

Publication	Sample size			Age (years)	Pre-R BMI (kg/m²)	Year of study	Location of study	Fasting duration (hours)	Timing of measurements (relative to the Ramadan fast)		
Aksungar 2005 (42)	12	12	24	21-35			Istanbul, Turkey	15	7 days before	Day 21	20 days after
Al-Numair 2006 (43)	45		45	30-45		2004	Buraidah, Saudi Arabia		1 day before	Day 28	
Dewanti 2006 (22)	37			17-62	24.2±3.2	2004	Indonesia	12*	3 days before	Day 21	
Lamine 2006 (44)	9	21	30	23.7±2.2		2003	Tunisia	12	21 days before	Day 21-28	21 days after
Subhan 2006 (45)	46		46	16-41	23.5±3.9	2001	Pakistan	10*	Pre-Ramadan	Ramadan	
Ziaee 2006 [M] (23)		39	80	18-29	23.1±6.0	2002	Iran	10.5*	3 days before	Day 26	
Ziaee 2006 [F] (23)	41				21.3±1.8						
Al-Hourani 2007 (46)			47	18-29	22.2±3.1	2004	Jordan	12	7 days before	Day 25-28	
Mansi 2007 (47)	42		42	21.3±1.6		2006	Jordan	12*	1 day before	Day 21-28	
Moosavi 2007 [M] (48)	77		117	26.2-29.6		1999-2000	Tehran, Iran	10*	10 days before	Second half of Ramadan	
Moosavi 2007 [F] (48)		40									
Souissi 2007 (49)	20		20	22.6±1.3		2005	Tunis, Tunisia	16	7 days before	Day 21-28	14 days after
Haouari 2008 (19)	36		36	24±1.6			Tunis, Tunisia	12	7 days before	Day 21	
Ibrahim 2008 (50)	9	5	14	25-58	24.6±3.8		UAE	16	2 days before	Day 28	
Stannard 2008 (51)	8		8	21-41		2000	Sydney, Australia	14.5	1-7 days before	Day 25-28	
Lamri-Senhadji2009 [M](20)	24		46	24±3			Algeria	14*	15 Days before	Day 28	15 days after
Lamri-Senhadji 2009[F](20)		22									
Pathan 2010 (52)	30		30	25-35			Maharashtra, India	12	1 day before	1 day after	
Assadi 2011 (53)	58		58	40.7±7.1			Iran		Day 1	Last day	
Trabelsi 2011 (24)	10		10	26.6±3	24.6±1.4	2010	Tunisia	15	3 days before	Day 29	21 days after
Ünalacak 2011 [Ob] (13)	10		20	27.4±5.2	28.1±2.1	2007	Turkey	12*	Pre-Ramadan	Post-Ramadan	
Ünalacak 2011 [Nw] (13)	10				23.7±1.4						
Faris 2012a (54)	21	29	50	18-51	26.3±5.0	2009	Rusaifa, Jordan	14.5	7 days before	Day 21	28 days after
Faris 2012b (55)	23	27	50	18-51	26.4±5.0		Rusaifa, Jordan	14.5	7 days before	Day 21	28 days after
Hajek 2012 (56)			87	34		2010	East London, UK	16	1-7 days before	1-7 days after	28 days after

Publication	Sample size			Age (years)	Pre-R BMI (kg/m²)	Year of study	Location of study	Fasting duration (hours)	Timing of measurements (relative to the Ramadan fast)		
Khattak 2012 [M, Nw] (14)	10		20		21.8±1.6		Malaysia	12*	Day1	Day 21	
Khattak 2012 [M, Ob] (14)					32.5±5.1						
Khattak 2012 [F, Nw] (14)		10			23.9±6.6						
Khattak 2012 [F, Ob] (14)					34.5±2.4						
Shehab 2012 [M] (57)	42		60	38.7±10.5	28.1±4.4		UAE		Day 1	Day 28	28 days after
Shehab 2012 [F] (57)		18			27.2±5.5						
Racinais 2012 (58)	11		11	31±3		2007	Qatar	11.5	7 days before	Day 21-28	Approx 30 days after
Agoumi 2013 (59)	22	33	55	18-70	29.5±6.0	2012	Barcelona, Spain	14*	10 days before	Day 28	
Develioglu 2013 (60)	35		35	20-59	25.5±3.5	2012	Turkey	12*	7 days before	Day 21-28	
Haouari-Oukerro 2013 (61)	38		38	18-23	23.3±0.3	2005	Tunisia	12	Just before	Day 27	
Norouzy 2013[M,18-35](62)	31		240	18-35	26.4±0.5	2008	Mashhad, Iran	14	1-7 days before	1-7 days after	
Norouzy 2013[F,18-35](62)		51			24.1±0.6						
Norouzy 2013[M,36-70](62)	127			36-70	26.8±0.3						
Norouzy 2013[F,36-70](62)		31			27.7±0.8						
Rohin 2013 [Nw] (15)			46	25-40		2012	Gong Badak, Malaysia	12*	7 days before	Day 14-21	28 days after
Rohin 2013 [Ow] (15)											
Rohin 2013 [Ob] (15)											
Sayedda 2013 (63)	20		20	19-32		2012	Uttar Pradesh, India	15	2 days before	Day 29	
Akaberi 2014 (64)			43	29.7±4.5		2011	Iran	16	1 day before	1 day after	
Feizollahzadeh 2014 (65)	7		7	30-70	27.98±1.38	2012	Iran	14.5	Pre Ramadan	Post Ramadan	
Cansel 2014 (66)	24	16	40	29.3±5.9	22.6±11.3	2010	Turkey	17	Before onset	After	
Celik 2014 [Nw] (16)	15		42	35±8.9	22.9±1.6	2012	Kahramamaras, Turkey	15.5	Day 1	Day 28	
Celik 2014 [Ow] (16)	17				27.2±1.6						
Celik 2014 [Ob] (16)	10				33.5±4.4						
McNeil 2014 [Nw] (17)	10		20	20-35	24.4±1.9	2008	Ottawa, Canada	12*	Before onset	Day 21	21-28 days after

Publication	Sample size			Age (years)	Pre-R BMI (kg/m²)	Year of study	Location of study	Fasting duration (hours)	Timing of measurements (relative to the Ramadan fast)		
McNeil 2014 [Ob] (17)	10				34.8±3.7						
Gnanou 2015 (67)	20		20	19-23	22.2±2.2		Malaysia	12	1-7 days before	Day 21-28	
Soori 2015 (68)	35		35	22-64		2014	Iran	14*		Last week	After
Lopez-Bueno 2015 (69)		62	62	18-61	26.3±5.8	2012+2013	Melilla, Spain	14*	7 days before	Day 21-28	
Talib 2015 (70)	45		45	27-56	30.2±4.9		Qatar		7 days before	Day 29, 30	
Sarici 2016 [Morning] (71)			29	38.1±10.2		2013	Istanbul, Turkey	14*		During	28 days after
Syam 2016 (26)	7	36	43	34.2±11.3	23.7±4.0	2013	Indonesia	12*	Day 1	Day 28	28-35 days after
Sezen 2016 (72)	70		70	37±7	27.9±0.6	2015	Turkey	16.5	1-7 days before	1-7 days after	
Alsubheen 2017 (73)	9		9	32.2±7.8	26.5±5.0	2015	Canada	15*	10 days before	Day 30	
Khan 2017 (74)	18	17	35	21.66±0.68		2011	Pakistan	15	1-10 days before	day 20-29	20-30 days after
Kiyani 2017 (75)	50	30	80	20.5		2013	Pakistan	13.5*	Pre-Ramadan	End Ramadan	
Latiri 2017 (76)	29		29	27 ± 5.39	27.1 ± 4.31	2014	Tunisia	16	7 days before	Day 16-18	14 days after
Malekmakan 2017 (77)	49	44	93	37.2 ± 7.9	26.1 ± 3.3	2015	Iran	14.5*	1-5 days before	1-3 days after	
Norouzy 2017 (78)	6	6	12	54.6 ± 4		2011	Iran	15.5	Before Ramadan	Last 10 days	30 days after
Nugraha 2017 (79)	25		25	26.12 ± 4.9	24.78 ± 3.65	2015	Germany	19	7 days before	Days 28-30	
Ongsara 2017 [M] (80)	21		65	20.86±1.35	22.94±4.12	2015	Thailand	12.5*	1st day of Ramadan	Last day of Ramadan	30 days after
Ongsara 2017 [F] (80)		44		20.8 ± 1.05	20.79±3.05						
Almeneessier 2017 (81)	8		8	26.6±4.9	23.7±3.5	2011	Saudi Arabia	13.5*	0-7 days before	Day 14	
Almeneessier 2018 (82)	8		8	25.4 ± 3.5	24.4 ± 3.8	2016	Saudi Arabia	13.5*	0-7 days before	Day 14	

Data for age and BMI are means ± standard deviations, or range. F = female; M = male; Nw = normal weight; Ow = overweight; Ob = obese; N-Ob = non-obese; Pre-R = before Ramadan; Post-R = end of Ramadan; Follow-up = 2-5 weeks after Ramadan; BMI = body mass index; * = fasting time not given in study but inferred based on year and location of study.

Table S1b. Details of weight and body composition from all included publications.

Publication	Weight (kg)			Method of body composition measurement	Fat percentage (%)			Absolute fat mass (kg)			Fat-free mass (kg)		
	Pre-R	Post-R	Follow-up		Pre-R	Post-R	Follow-up	Pre-R	Post-R	Follow-up	Pre-R	Post-R	Follow-up
Fedail 1982 (28)	69.5±2.3	67.7±2.4											
Husain 1987 [M] (29)	62.9±5.9		62.6±6.3										
Husain 1987 [F] (29)	49.9±6.5		49.2±6.6										
Takruri 1989 [Ow] (11)	74.8±12.3	72.2±12.1											
Takruri 1989 [Nw] (11)	62.0±7.8	60.0±7.5											
Azizi 1992 (30)	65.4±9.1	61.6±9	64.8±8.5										
Sweileh 1992 (31)	72.1±10.8	70.2±11.5		Hydrodensitometry	22.7±0.6	20.5±6.8					55.5±6.4	55.9±7.1	
Maislos 1993 (32)		68.0±17.0	68.2±16.0										
El-Ati 1995 (33)	59.3±5.2	58.9±4.8	58.6±5.6	Skin caliper: Harpended caliper				16.7±5.2	16.2±5.2	16.1±4.8	43.3±2.4	43.2±2.8	43.1±2.8
Adlouni 1998 (34)	69.7±10.8	67.8±10.7	69.1±10.1										
Bilto 1998 (35)	72.0±12.6	70.8±13.0	71.1±12.9										
Finch 1998 (36)	71.0±12.0	70.7±6.9	70.8±5.7										
Kayikcioglu 1998 (37)	71.7±7.3	70.7±7.0											
Fakhrzadeh 2003 [M] (38)	65.0±8.4	63.7±7.9											
Fakhrzadeh 2003 [F] (38)	60.7±13.0	60.3±14.2											
Kassab 2003 [N-Ob] (12)	72.5±13.3	71.7±15.3	72.3±12.8	Bioelectrical impedance	29.1±7.7	29.5±8.1	29.0±8.7	17.7±6.1	17.5±6.6	17.6±7.1			
Kassab 2003 [Ob] (12)	89.0±22.0	88.6±22.5	88.6±22.5		38.8±4.7	37.4±7.2	38.9±4.7	34.9±10.2	31.3±8.5	34.8±10.6			
Kassab 2004 (39)	80.8±9.5	80.1±13.6	80.5±12.2	Bioelectrical impedance: OMRON BF 302	32.8±8.8	32.5±9.5	32.9±11.5	23.9±12.2	22.4±11.5	23.7±12.9			
Rahman 2004 (40)	64.1±7.8	62.1±8.1	63.1±7.8										
Saleh 2004 (18)	98.9±19.2	97.4±19.9	97.7±23.5	Equation from Filer et al. (1998)	49.6±10.8	44.7±6.4	45.7±7.8						
Yucel 2004 [M] (41)	75.9±15.0	75.8±15											
Yucel 2004 [F] (41)	62.1±10.4	60.6±9.5											
Aksungar 2005 (42)	82.2±5.5	82.5±6.1	82.4±5.2										

Publication	Weight (kg)			Method of body composition measurement	Fat percentage (%)			Absolute fat mass (kg)			Fat-free mass (kg)		
Al-Numair 2006 (43)	85.5±3.9	83.2±3.3											
Dewanti 2006 (22)	64.5±11.1	63.0±10.8											
Lamine 2006 (44)	61.3±10.2	61.7±10.4	62.1±10.5										
Subhan 2006 (45)	70.5±14.9	70.0±14.9	70.8±14.5										
Ziaee 2006 [M] (23)	68.7±12.1	67.5±10.8											
Ziaee 2006 [F] (23)	55.7±5.8	54.6±5.9											
Al-Hourani 2007 (46)	57.5±8.2	56.9±7.4		Bioelectrical impedance: Tanita BC-532 total inner scan	24.9±6.6	24.5±6.8					40.5±3.4	40.4±2.2	
Mansi 2007 (47)	76.6±9.5	72.7±9.2	73.6±8.8										
Moosavi 2007 [M] (48)	70.4±10.3	69.5±10.4	69.8±10.5										
Moosavi 2007 [F] (48)	62.2±8.6	61.1±8.7	61.8±8.5										
Souissi 2007 (49)	71.3±2.33	71.7±7.2	71.7±7.5										
Haouari 2008 (19)	70.9±2.1	70.5±2.1											
Ibrahim 2008 (50)	70.5±10.9	69.1±10.6		Bioelectrical impedance: TANITA body composition analyser				21.7±10.5	22.1±10.2		48.4±11.3	47.3±10.5	
Stannard 2008 (51)	71.1±6.9	69.8±7.3		Hydrodensitometry	12.9±3.5	12.2±3.2		9.1±2.1	8.4±1.7		62.0±7.7	62.1±7.9	
Lamri-Senhadji2009 [M](20)	70.0±42.2	70.0±46.9	70.0±51.6										
Lamri-Senhadji 2009[F](20)	53.0±39.2	52.0±44.1	54.0±44.1										
Pathan 2010 (52)	61.9±11.4	60.6±10.7											
Assadi 2011 (53)	78.6±12.1	77.2±12.7											
Trabelsi 2011 (24)	79.2±3.0	77.7±3.0	78.7±2.7	Skin caliper: Harpended caliper	19.4±1.3	18.2±0.7	18.9±1.5				63.8±3.0	63.6±2.7	63.9±3.1
Ünalacak 2011 [Ob] (13)	83.2±8.4	80.3±7.9											
Ünalacak 2011 [Nw] (13)	71.9±5.9	71.1±6.0											
Faris 2012a (54)	71.8±13.4	70.6±13.2	71.9±13.5	Bioelectrical impedance: TANITA body composition analyser	24.1±12.6	20.4±11.3	30.4±11.3						
Faris 2012b (55)	72.5±14.0	71.7±13.7	72.6±14.0	Bioelectrical impedance: GIMA body fat analyser	24.3±12.0	21.2±11.5	30.3±10.7						
Hajek 2012 (56)	71.2±12.4	70.6±12.5	71.1±12.5										

Publication	Weight (kg)			Method of body composition measurement	Fat percentage (%)			Absolute fat mass (kg)			Fat-free mass (kg)		
Khattak 2012 [M, Nw] (14)	60.2±5.6	53.5±4.6		Bioelectrical impedance: MALTRON BioScan	13.7±3.4	14.1±2.9							
Khattak 2012 [M, Ob] (14)	102.8±6.1	87.0±10.8			33.8±3.9	33.1±2.1							
Khattak 2012 [F, Nw] (14)	59.3±6.2	51.9±4.7			26.4±9.8	26.8±10.0							
Khattak 2012 [F, Ob] (14)	100.7±7.5	85.3±11.3			44.4±1.3	43.8±0.9							
Shehab 2012 [M] (57)	82.9±14.6	81.8±14.6	82.4±15.1										
Shehab 2012 [F] (57)	68.5±15.1	67.9±15.3	68.3±14.9										
Racinais 2012 (58)	70.9±10.8	70.0±8.9	70.8±8.6	Bioelectrical impedance: Quantum II				14.9±6.3	14.1±4.8	15.4±4.6	55.7±6.9	55.9±6.5	55.5±5.8
Agoumi 2013 (59)	77.8±15.3	76.7±15.1	76.8±14.8	Bioelectrical impedance: Medisana electronic scale	30.3±11.0	30.5±12.0	31.1±11.6						
Develioglu 2013 (60)	77.2±11.4	76.0±11.7											
Haouari-Oukerro 2013 (61)	70.0±0.7	68.7±0.6		Bioelectrical impedance: Tanita TBF-401 A”				10.1±0.3	9.1±0.3		59.6±0.5	58.9±0.5	
Norouzy 2013[M,18-35](62)	76.8±9.5	75.1±9.5		Bioelectrical impedance: Jawon Plus Avis 333				20.6±5.0	19.7±5.6		56.5±6.1	55.3±5.6	
Norouzy 2013[F,18-35](62)	59.9±12.1	59.1±12.1						19.1±7.1	18.7±7.1		41.0±5.7	40.5±5.0	
Norouzy 2013[M,36-70](62)	76.5±11.3	75.3±11.3						20.6±5.6	20.1±5.6		55.9±6.8	55.4±6.8	
Norouzy 2013[F,36-70](62)	67.2±10.6	66.7±11.1						23.8±6.7	24.0±6.7		43.5±5.0	42.6±5.0	
Rohin 2013 [Nw] (15)	53.6±7.8	52.4±7.6	52.8±7.8	Bioelectrical impedance: Omron Karada Scan HBF-36	29.8±6.1	28.6±5.5	28.7±5.5						
Rohin 2013 [Ow] (15)	71.2±7.1	69.9±7.2	70.2±7.2		31.1±4.5	30.7±4.6	30.5±4.9						
Rohin 2013 [Ob] (15)	82.6±9.3	80.9±10.0	80.4±10.8		35.5±4.3	34.4±4.7	34.77±4.5						
Sayedda 2013 (63)	71.1±8.2	68.9±8.0											
Akaberi 2014 (64)	71.6±12.1	70.2±11.5											
Feizollahzadeh 2014 (65)	79.8±8.95	77.9±8.93											
Cansel 2014 (66)	61.8±7.5	62.3±7.1											
Celik 2014 [Nw] (16)	69.1±7.3	67.8±6.9											
Celik 2014 [Ow] (16)	81.8±6.8	80.5±7											
Celik 2014 [Ob] (16)	101.1±15.1	98.3±13.9											

Publication	Weight (kg)			Method of body composition measurement	Fat percentage (%)			Absolute fat mass (kg)			Fat-free mass (kg)		
McNeil 2014 [Nw] (17)	72.8±9.2	71.8±9.4	72.1±9.6	Bioelectrical impedance: Tanita HR-100				17.6±4.2	17.6±4.5	17.4±4.9	55.1±5.9	54.1±5.8	54.6±5.7
McNeil 2014 [Ob] (17)	107.9±15.7	105.3±15.4	105.0±15.1					39.1±10.3	37.7±19.6	37.8±9.7	68.2±9.4	67.1±9.5	67.0±8.6
Gnanou 2015 (67)	63.1±8.19.0	61.6±8.11.0											
Soori 2015 (68)		77.3±10.6	80.75±10.9										
Lopez-Bueno 2015 (69)	67.2±14.1	66.1±15.0		Bioelectrical impedance: TANITA SC-330	32.10±9.4	31.4±9.5							
Talib 2015 (70)	94.7±17.3	94.0±17.2											
Sarici 2016 [Morning] (71)		72.7±12.7	73.5±13.5										
Syam 2016 (26)	59.8±11.3	59.9±11.2	58.7±10.6	Bioelectrical impedance: GAIA 359 PLUS				17.0±6.4	16.5±6.3				
Sezen 2016 (72)				Bioelectrical impedance: Segmental body composition analyser	22.5 ± 5.0	21.7±4.6		18.5±5.2	17.6±4.7		63.0±6.9	62.7±6.7	
Alsubheen 2017 (73)	82.9±15.8			Hydrodensitometry	29·6±8.4			25·7±10.8			57·3±7.4		
Khan 2017 (74)	60.5±14.7	60.5±15.0	60.2±14.5										
Kiyani 2017 (75)	62.7 ± 8.8	62.3±9.0											
Latiri 2017 (76)	81.6±15.29	80.8±15.62	81.2±15.6										
Malekmakan 2017 (77)	71.6±12.4	70.4±12.0											
Norouzy 2017 (78)	67.4±12	67.5±12	67.2±11										
Nugraha 2017 (79)	77.8±12.3	76.04±12.7		Bioelectrical impedance: InBody 230	20.92 ± 7.05	20.5±6.3		16.7±7.4	17.3±9.7		61.2 ± 8.6	60.1±8.8	
Ongsara 2017 [M] (80)	64.6±15.3	63.7±15.4	64.7±16.0	Bioelectrical impedance: TANITA SC-330	18.17±7.35	17.6±6.5	21.0±8.9	12.7±7.9	12.2±7.5	14.9±10.4	52.5±8.4	52.2±8.5	50.5±7.9
Ongsara 2017 [F] (80)	51.4±9.1	51.0±9.9	51.3±9.1		26.58±5.79	25.2±6.5	26.4±5.8	14.1±5.6	13.1±5.6	13.9±5.6	37.3±4.1	37.4±4.1	37.2±4.1
Almeneessier 2017 (81)	69.1±8.4	66.3±12.3											
Almeneessier 2018 (82)	71.2 ± 7.9	69.6±10.8											

Data are means ± standard deviations. F = female; M = male; Nw = normal weight; Ow = overweight; Ob = obese; N-Ob = non-obese; Pre-R= before Ramadan; Post-R = end of Ramadan; Follow-up = 2-5 weeks after Ramadan.

Table S1c. Details of physical activity for all included publications.

Publication	Method of physical activity measurement	Maximum effort physical activity			Daily physical activity		
		Pre-R	Post-R	Follow-up	Pre-R	Post-R	Follow-up
Fedail 1982 (28)							
Husain 1987 [M] (29)							
Husain 1987 [F] (29)							
Takruri 1989 [Ow] (11)							
Takruri 1989 [Nw] (11)							
Azizi 1992 (30)							
Sweileh 1992 (31)	VO_2 max using Quinton treadmill and Beckman medical gas analyzers (ml/kg/min)	39.0±4.5	40.5±5.1				
Maislos 1993 (32)							
El-Ati 1995 (33)							
Adlouni 1998 (34)							
Bilto 1998 (35)							
Finch 1998 (36)							
Kayikcioglu 1998 (37)							
Fakhrzadeh 2003 [M] (38)							
Fakhrzadeh 2003 [F] (38)							
Kassab 2003 [N-Ob] (12)							
Kassab 2003 [Ob] (12)							
Kassab 2004 (39)							
Rahman 2004 (40)							
Saleh 2004 (18)							
Yucel 2004 [M] (41)							
Yucel 2004 [F] (41)							
Aksungar 2005 (42)							

Publication	Method of physical activity measurement	Maximum effort physical activity			Daily physical activity		
Al-Numair 2006 (43)							
Dewanti 2006 (22)							
Lamine 2006 (44)							
Subhan 2006 (45)							
Ziaee 2006 [M] (23)							
Ziaee 2006 [F] (23)							
Al-Hourani 2007 (46)							
Mansi 2007 (47)							
Moosavi 2007 [M] (48)							
Moosavi 2007 [F] (48)							
Souissi 2007 (49)	Mean power output during Wingate test (W/kg)	9.0±1.9	8.6±2.4	8.9±2.2			
Haouari 2008 (19)							
Ibrahim 2008 (50)							
Stannard 2008 (51)							
Lamri-Senhadji2009 [M](20)							
Lamri-Senhadji 2009[F](20)							
Pathan 2010 (52)							
Assadi 2011 (53)							
Trabelsi 2011 (24)							
Ünalacak 2011 [Ob] (13)							
Ünalacak 2011 [Nw] (13)							
Faris 2012a (54)							
Faris 2012b (55)							
Hajek 2012 (56)							
Khattak 2012 [M, Nw] (14)							
Khattak 2012 [M, Ob] (14)							
Khattak 2012 [F, Nw] (14)							

Publication	Method of physical activity measurement	Maximum effort physical activity			Daily physical activity		
Khattak 2012 [F, Ob] (14)							
Shehab 2012 [M] (57)							
Shehab 2012 [F] (57)							
Racinais 2012 (58)	Maximal voluntary contraction of quadriceps (Nm)	244.6±34.0	244.3±26.5	252.5±30.6			
Agoumi 2013 (59)							
Develioglu 2013 (60)							
Haouari-Oukerro 2013 (61)							
Norouzy 2013[M,18-35](62)							
Norouzy 2013[F,18-35](62)							
Norouzy 2013[M,36-70](62)							
Norouzy 2013[F,36-70](62)							
Rohin 2013 [Nw] (15)							
Rohin 2013 [Ow] (15)							
Rohin 2013 [Ob] (15)							
Sayedda 2013 (63)							
Akaberi 2014 (64)							
Feizollahzadeh 2014 (65)							
Cansel 2014 (66)							
Celik 2014 [Nw] (16)							
Celik 2014 [Ow] (16)							
Celik 2014 [Ob] (16)							
McNeil 2014 [Nw] (17)							
McNeil 2014 [Ob] (17)							
Gnanou 2015 (67)							
Soori 2015 (68)							
Lopez-Bueno 2015 (69)							
Talib 2015 (70)							

Publication	Method of physical activity measurement	Maximum effort physical activity			Daily physical activity		
Sarici 2016 [Morning] (71)							
Syam 2016 (26)							
Sezen 2016 (72)							
Alsubheen 2017 (73)	Physical activity tracker (Vivofit) (Steps/day)				6368.0±1556.0	6714.0±1627.0	
Khan 2017 (74)	Physical activity = MET (metabolic energy turnover) value × duration (min per session) × frequency (activity per week).				172.5±241.9	136.1±165.5	236.3±281.2
Kiyani 2017 (75)							
Latiri 2017 (76)							
Malekmakan 2017 (77)							
Norouzy 2017 (78)							
Nugraha 2017 (79)	SF-12 questionnaire for physical health				52.6±5.4	50.3±7.5	
Ongsara 2017 [M] (80)							
Ongsara 2017 [F] (80)							
Almeneessier 2017 (81)							
Almeneessier 2018 (82)							

Data are means ± standard deviations. F = female; M = male; Nw = normal weight; Ow = overweight; Ob = obese; N-Ob = non-obese; Pre-R= before Ramadan; Post-R = end of Ramadan; Follow-up = 2-5 weeks after Ramadan.

Publication	Difference in means	Lower limit	Upper limit	p-Value
	Statistics for each study			
Fedail 1982 (28)	-1.80	-2.40	-1.20	0.000
Takruri 1989 [Ow] (11)	-2.62	-4.37	-0.87	0.003
Takruri 1989 [Nw] (11)	-1.99	-3.54	-0.44	0.012
Azizi 1992 (30)	-3.80	-7.54	-0.06	0.046
Sweileh 1992 (31)	-1.92	-7.17	3.33	0.473
El-Ati 1995 (33)	-0.40	-1.96	1.16	0.615
Adlouni 1998 (34)	-1.78	-4.14	0.58	0.140
Bilto 1998 (35)	-1.20	-3.62	1.22	0.332
Finch 1998 (36)	-0.30	-2.66	2.06	0.803
Kayikcioglu 1998 (37)	-0.90	-2.47	0.67	0.261
Fakhrzadeh 2003 [M] (38)	-1.20	-2.63	0.23	0.101
Fakhrzadeh 2003 [F] (38)	-0.40	-3.06	2.26	0.768
Kassab 2003 [N-Ob] (12)	-0.80	-4.35	2.75	0.659
Kassab 2003 [Ob] (12)	-0.40	-6.91	6.11	0.904
Kassab 2004 (39)	-0.70	-3.08	1.68	0.565
Rahman 2004 (40)	-1.98	-4.18	0.22	0.078
Saleh 2004 (18)	-1.50	-3.89	0.89	0.219
Yucel 2004 [M] (41)	-0.16	-4.21	3.89	0.938
Yucel 2004 [F] (41)	-1.49	-4.51	1.53	0.334
Aksungar 2005 (42)	0.23	-1.24	1.70	0.760
Al-Numair 2006 (43)	-2.30	-2.99	-1.61	0.000
Dewanti 2006 (22)	-1.50	-3.73	0.73	0.188
Lamine 2006 (44)	0.40	-1.93	2.73	0.737
Subhan 2006 (45)	-0.52	-3.25	2.21	0.709
Ziaee 2006 [M] (23)	-1.20	-3.51	1.11	0.308
Ziaee 2006 [F] (23)	-1.10	-2.23	0.03	0.057
Al-Hourani 2007 (46)	-0.60	-2.03	0.83	0.410
Mansi 2007 (47)	-3.98	-5.37	-2.59	0.000
Moosavi 2007 [M] (48)	-0.90	-2.36	0.56	0.228
Moosavi 2007 [F] (48)	-1.10	-2.79	0.59	0.202
Souissi 2007 (49)	0.43	-1.99	2.85	0.727
Haouari 2008 (19)	-0.40	-0.83	0.03	0.071
Ibrahim 2008 (50)	-1.40	-4.96	2.16	0.441
Stannard 2008 (51)	-1.27	-4.39	1.85	0.424
Lamri-Senhadji 2009 [M] (20)	0.00	-11.92	11.92	1.000
Lamri-Senhadji 2009 [F] (20)	-1.00	-11.70	9.70	0.855
Pathan 2010 (52)	-1.34	-3.85	1.17	0.296
Assadi 2011 (53)	-1.37	-3.40	0.66	0.185
Trabelsi 2011 (24)	-1.50	-2.68	-0.32	0.012
Ünalacak 2011 [Ob] (13)	-2.90	-6.11	0.31	0.076
Ünalacak 2011 [Nw] (13)	-0.80	-3.13	1.53	0.502
Faris 2012a (54)	-1.24	-3.57	1.09	0.298
Faris 2012b (55)	-0.80	-3.23	1.63	0.519
Hajek 2012 (56)	-0.62	-2.27	1.03	0.463
Khattak 2012 [M, Nw] (14)	-6.70	-9.67	-3.73	0.000
Khattak 2012 [M, Ob] (14)	-15.80	-21.87	-9.73	0.000
Khattak 2012 [F, Nw] (14)	-7.40	-11.03	-3.77	0.000
Khattak 2012 [F, Ob] (14)	-15.40	-20.97	-9.83	0.000
Shehab 2012 [M] (57)	-1.10	-3.89	1.69	0.440
Shehab 2012 [F] (57)	-0.60	-5.04	3.84	0.791
Racinais 2012 (58)	-0.90	-4.73	2.93	0.645
Agoumi 2013 (59)	-1.09	-3.63	1.45	0.401
Develioglu 2013 (60)	-1.20	-3.62	1.22	0.331
Haouari-Oukerro 2013 (61)	-1.30	-1.43	-1.17	0.000
Norouzy 2013 [M, 18-35] (62)	-1.70	-3.81	0.41	0.114
Norouzy 2013 [F, 18-35] (62)	-0.80	-2.91	1.31	0.457
Norouzy 2013 [M, 36-70] (62)	-1.20	-2.44	0.04	0.058
Norouzy 2013 [F, 36-70] (62)	-0.50	-2.93	1.93	0.686
Rohin 2013 [Nw] (15)	-1.27	-3.46	0.92	0.256
Rohin 2013 [Ow] (15)	-1.29	-3.38	0.80	0.227
Rohin 2013 [Ob] (15)	-1.66	-5.65	2.33	0.415
Sayedda 2013 (63)	-2.18	-4.43	0.07	0.057
Akaberi 2014 (64)	-1.40	-3.64	0.84	0.220
Cansel 2014 (66)	0.50	-8.58	9.58	0.914
Celik 2014 [Nw] (16)	-1.30	-3.58	0.98	0.264
Celik 2014 [Ow] (16)	-1.30	-3.38	0.78	0.220
Celik 2014 [Ob] (16)	-2.80	-8.53	2.93	0.338
Feizollahzadeh 2014 (65)	-1.84	-6.03	2.35	0.389
McNeil 2014 [Nw] (17)	-1.00	-4.65	2.65	0.591
McNeil 2014 [Ob] (17)	-2.60	-8.70	3.50	0.403
Gnanou 2015 (67)	-1.52	-3.78	0.74	0.187
Lopez-Bueno 2015 (69)	-1.10	-3.40	1.20	0.348
Talib 2015 (70)	-0.67	-3.86	2.52	0.680
Syam 2016 (26)	0.03	-2.09	2.15	0.978
Khan 2017 (74)	-0.03	-4.38	4.32	0.989
Kiyani 2017 (75)	-0.40	-1.96	1.16	0.616
Latiri 2017 (76)	-0.80	-4.36	2.76	0.660
Malekmakan 2017 (77)	-1.20	-3.36	0.96	0.277
Norouzy 2017 (78)	0.10	-5.97	6.17	0.974
Nugraha 2017 (79)	-1.78	-4.88	1.32	0.261
Ongsara 2017 [M] (80)	-0.90	-5.05	3.25	0.671
Ongsara 2017 [F] (80)	-0.46	-2.25	1.33	0.615
Almeneessier 2017 (81)	-2.80	-8.01	2.41	0.292
Almeneessier 2018 (82)	-1.60	-6.12	2.92	0.488
	-1.34	-1.61	-1.07	0.000

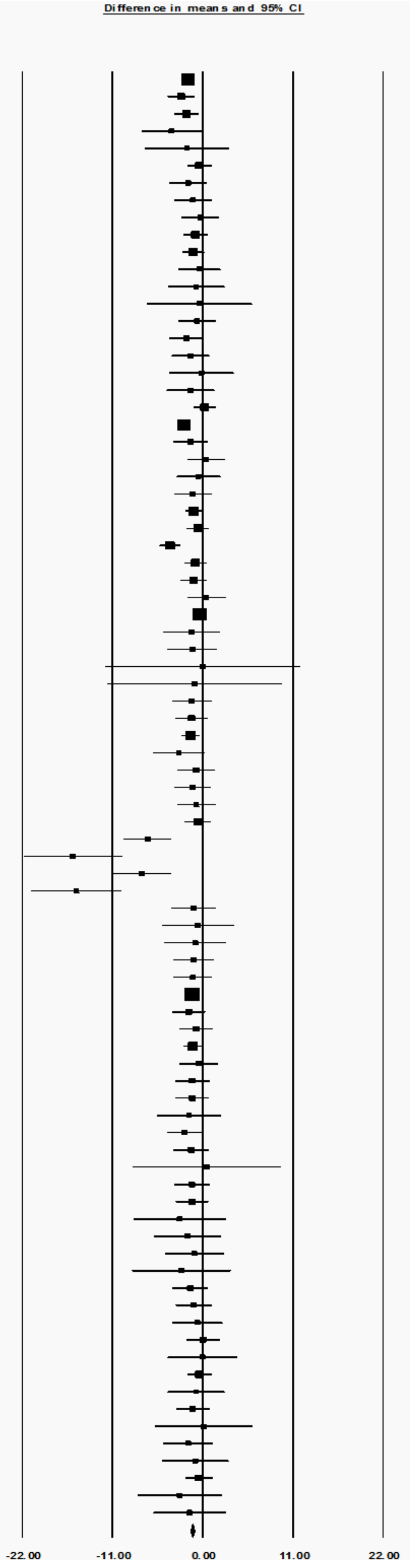

Figure S1. Weight was significantly decreased by Ramadan fasting. Change in weight (kg) between pre-Ramadan (pre-R) and the end of Ramadan (post-R). Heterogeneity statistics: $T = 0.586$, $p < 0.001$, $I^2 = 40.6\%$. F = female; M = male; Nw = normal weight; Ow = overweight; Ob = obese; N-Ob = non-obese; 18-35, 36-70 = age range in years.

Publication	Statistics for each study			
	Difference in means	Lower limit	Upper limit	p-Value
Husain 1987 [M] (29)	-0.30	-2.49	1.89	0.789
Husain 1987 [F] (29)	-0.70	-3.41	2.01	0.612
Azizi 1992 (30)	-0.60	-4.26	3.06	0.748
El-Ati 1995 (33)	-0.70	-2.38	0.98	0.415
Adlouni 1998 (34)	-0.51	-2.82	1.80	0.665
Finch 1998 (36)	-0.20	-2.72	2.32	0.877
Kassab 2003 [N-Ob] (12)	-0.20	-3.37	2.97	0.901
Kassab 2003 [Ob] (12)	-0.40	-6.91	6.11	0.904
Kassab 2004 (39)	-0.30	-2.42	1.82	0.781
Rahman 2004 (40)	-1.00	-3.15	1.15	0.362
Saleh 2004 (18)	-1.20	-3.93	1.53	0.388
Aksungar 2005 (42)	0.17	-1.18	1.52	0.806
Lamine 2006 (44)	0.80	-1.54	3.14	0.504
Mansi 2007 (47)	-3.00	-4.37	-1.63	0.000
Souissi 2007 (49)	0.37	-2.17	2.91	0.776
Lamri-Senhadji 2009 [M] (20)	0.00	-12.94	12.94	1.000
Lamri-Senhadji 2009 [F] (20)	1.00	-9.70	11.70	0.855
Trabelsi 2011 (24)	-0.50	-1.63	0.63	0.386
Faris 2012a (54)	0.10	-2.26	2.46	0.934
Faris 2012b (55)	0.10	-2.35	2.55	0.936
Hajek 2012 (56)	-0.12	-1.78	1.54	0.887
Shehab 2012 [M] (57)	-0.50	-3.34	2.34	0.730
Shehab 2012 [F] (57)	-0.20	-4.58	4.18	0.929
Racinais 2012 (58)	-0.10	-3.93	3.73	0.959
Rohin 2013 [Nw] (15)	-0.77	-2.99	1.45	0.497
Rohin 2013 [Ow] (15)	-0.95	-3.04	1.14	0.373
Rohin 2013 [Ob] (15)	-2.07	-6.34	2.20	0.342
McNeil 2014 [Nw] (17)	-0.70	-4.39	2.99	0.710
McNeil 2014 [Ob] (17)	-2.90	-8.95	3.15	0.347
Syam 2016 (26)	-1.13	-3.20	0.94	0.285
Khan 2017 (74)	-0.32	-4.60	3.96	0.883
Latiri 2017 (76)	-0.40	-3.96	3.16	0.826
Norouzy 2017 (78)	-0.20	-6.07	5.67	0.947
Ongsara 2017 [M] (80)	0.15	-4.09	4.39	0.945
Ongsara 2017 [F] (80)	-0.08	-1.78	1.62	0.926
	-0.59	-0.99	-0.20	0.003

Difference in means and 95% CI

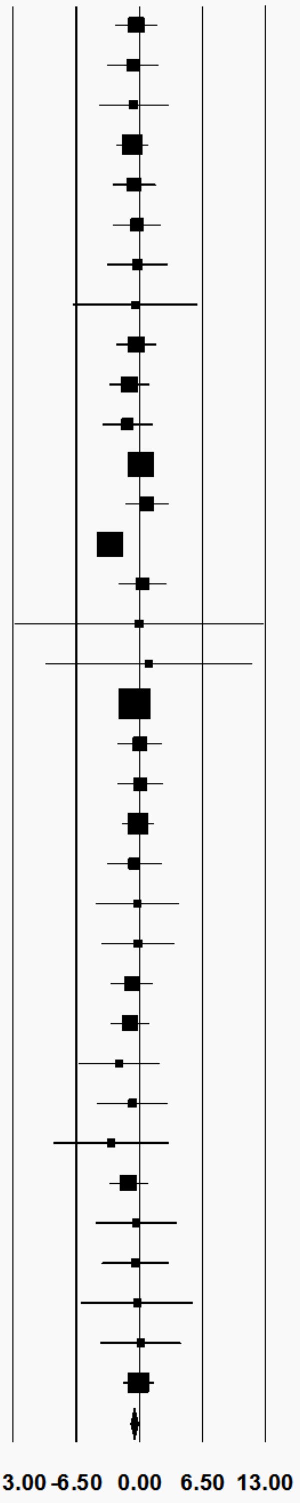

Figure S2. Weight was significantly reduced at 2-5 weeks after Ramadan fasting. Change in weight (kg) between pre-Ramadan (pre-R) and 2-5 weeks after Ramadan (follow-up). Heterogeneity statistics: $T = 0.000$, $p = 0.940$, $I^2 = 0.0\%$. F = female; M = male; Nw = normal weight; Ow = overweight; Ob = obese; N-Ob = non-obese.

Publication	Subgroup	Difference in means	Lower limit	Upper limit	p-Value
Takruri 1989 [Nw] (11)	Nw	-1.99	-3.54	-0.44	0.012
Kassab 2003 [N-Ob] (12)	Nw	-0.80	-4.35	2.75	0.659
Haouari 2008 (19)	Nw	-0.40	-0.83	0.03	0.071
Lamri-Senhadji 2009 [M] (20)	Nw	0.00	-11.92	11.92	1.000
Lamri-Senhadji 2009 [F] (20)	Nw	-1.00	-11.70	9.70	0.855
Ünalacak 2011 [Nw] (13)	Nw	-0.80	-3.13	1.53	0.502
Khattak 2012 [M, Nw] (14)	Nw	-6.70	-9.67	-3.73	0.000
Khattak 2012 [F, Nw] (14)	Nw	-7.40	-11.03	-3.77	0.000
Rohin 2013 [Nw] (15)	Nw	-1.27	-3.46	0.92	0.256
Celik 2014 [Nw] (16)	Nw	-1.30	-3.58	0.98	0.264
McNeil 2014 [Nw] (17)	Nw	-1.00	-4.65	2.65	0.591
		-2.06	-3.37	-0.74	0.002
Takruri 1989 [Ow] (11)	Ow/Ob	-2.62	-4.37	-0.87	0.003
Kassab 2003 [Ob] (12)	Ow/Ob	-0.40	-6.91	6.11	0.904
Saleh 2004 (18)	Ow/Ob	-1.50	-3.89	0.89	0.219
Ünalacak 2011 [Ob] (13)	Ow/Ob	-2.90	-6.11	0.31	0.076
Khattak 2012 [M, Ob] (14)	Ow/Ob	-15.80	-21.87	-9.73	0.000
Khattak 2012 [F, Ob] (14)	Ow/Ob	-15.40	-20.97	-9.83	0.000
Rohin 2013 [Ow] (15)	Ow/Ob	-1.29	-3.38	0.80	0.227
Rohin 2013 [Ob] (15)	Ow/Ob	-1.66	-5.89	2.57	0.442
Celik 2014 [Ow] (16)	Ow/Ob	-1.30	-3.38	0.78	0.220
Celik 2014 [Ob] (16)	Ow/Ob	-2.80	-8.53	2.93	0.338
McNeil 2014 [Ob] (17)	Ow/Ob	-2.60	-8.70	3.50	0.403
Feizollahzadeh 2014 (65)	Ow/Ob	-1.84	-6.03	2.35	0.389
		-3.53	-5.47	-1.60	0.000
		-2.52	-3.61	-1.44	0.000

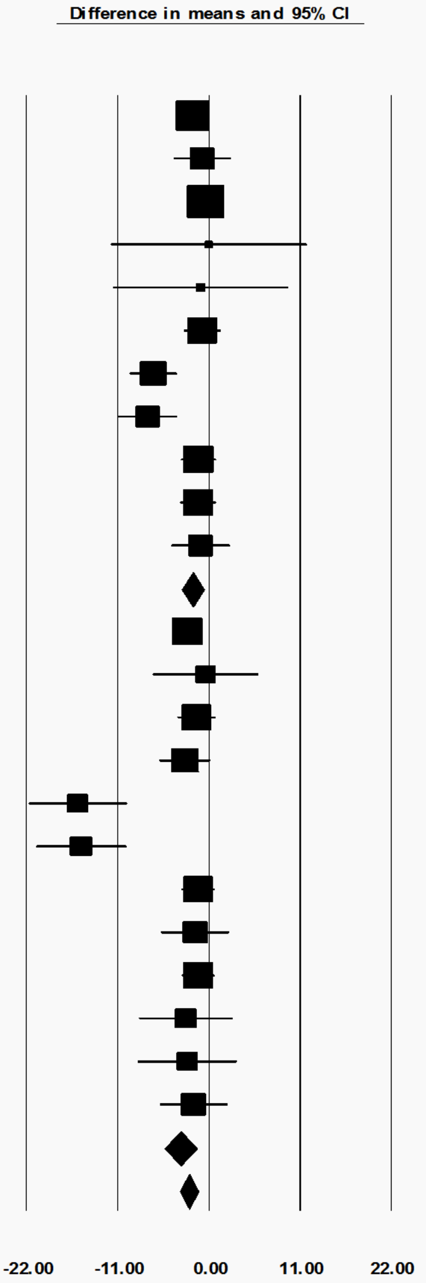

Figure S3. Weight was significantly decreased by Ramadan fasting in both the normal weight and the overweight/obese subgroup by the end of Ramadan. Change in weight (kg) as subdivided by BMI category (normal weight and overweight/obese), between pre-Ramadan (pre-R) and the end of Ramadan (post-R). There was no significant difference between normal weight and overweight/obese subgroups (p = 0.217). Heterogeneity statistics: normal weight T = 1.607, $p < 0.001$, I^2 = 70.4%; overweight/obese T = 2.738, $p < 0.001$, I^2 = 74.4%. F = female; M = male; Nw = normal weight; Ow = overweight; Ob = obese; N-Ob = non-obese.

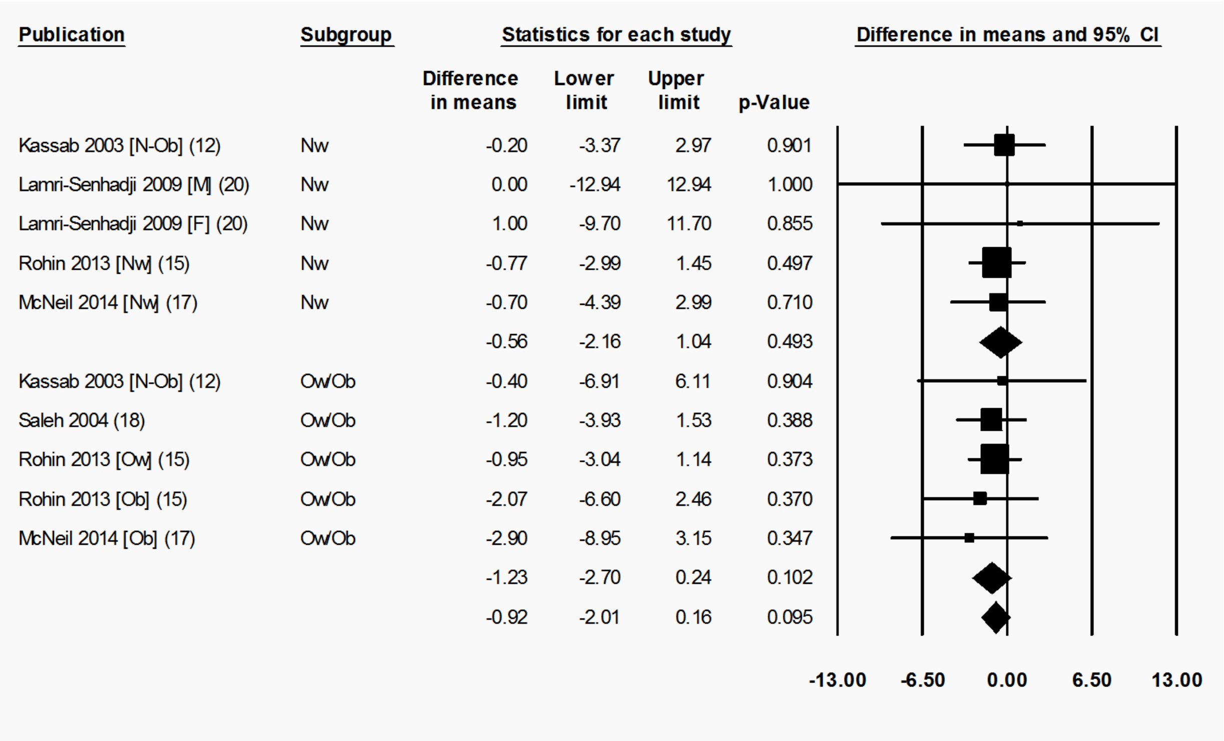

Figure S4. Weight was not significantly reduced at 2-5 weeks after Ramadan in the normal weight or the overweight/obese subgroup. Change in weight (kg) as subdivided by BMI category (normal weight and overweight/obese), between pre-Ramadan (pre-R) and 2-5 weeks after Ramadan (follow-up). There was no significant difference between normal weight and overweight/obese subgroups (p = 0.547). Heterogeneity statistics: normal weight T = 0.000, p = 0.996, I^2 = 0.0%; overweight/obese T = 0.000, p = 0.968, I^2 = 0.0%. F = female; M = male; Nw = normal weight; Ow = overweight; Ob = obese; N-Ob = non-obese.

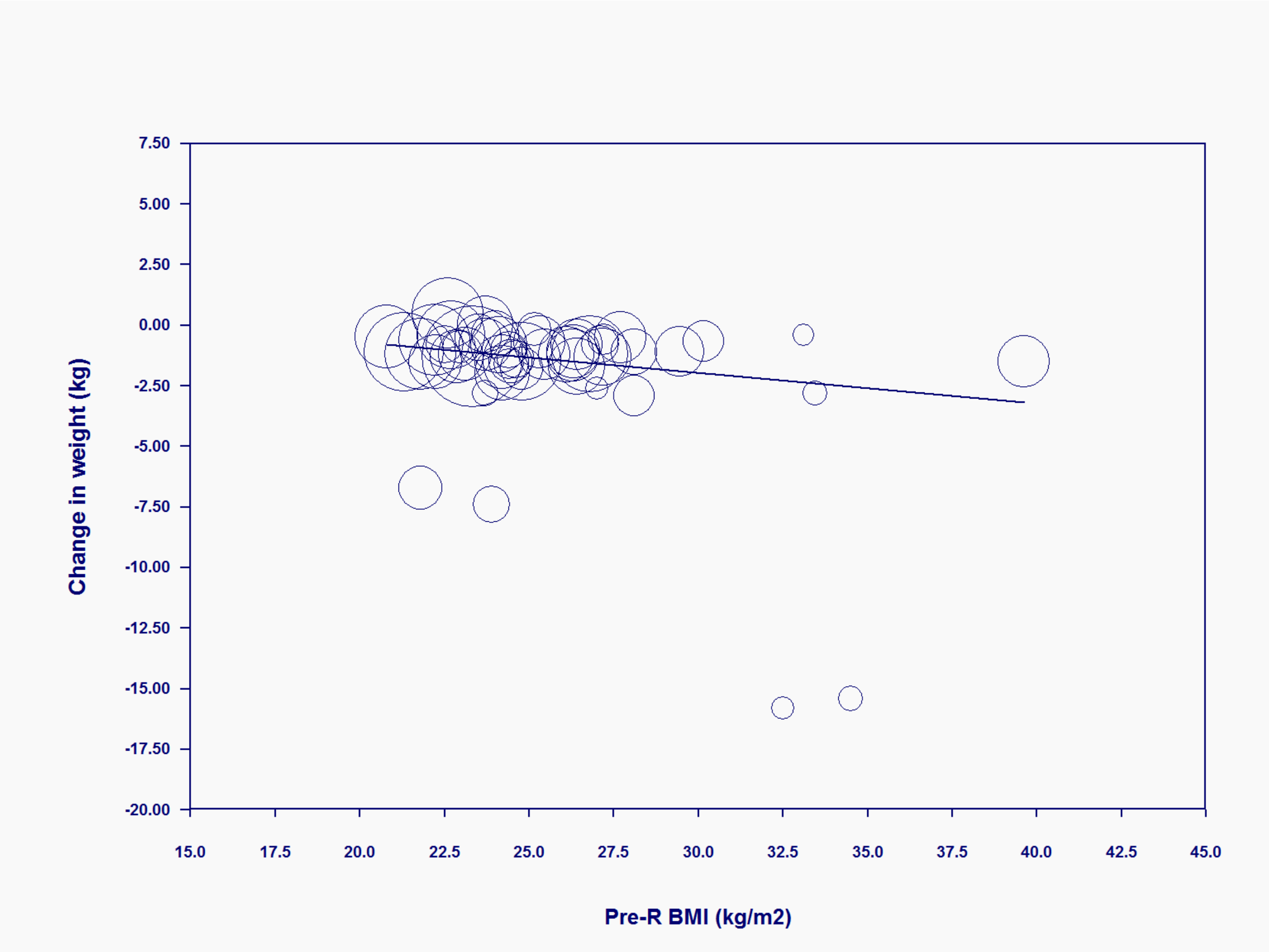

Figure S5. Significant correlation between BMI before Ramadan fasting and mean weight change. Scatterplot of the meta-regression analysis for the correlation between BMI measured pre-Ramadan (pre-R) and weight change from pre-R to the end of Ramadan (post-R) for each of the 51 comparison groups taken from publications that obtained both pre-R BMI and weight data at these two time points (pre- R and post-R). The area of each circle represents the percentage weight contributed by each study to the overall meta-regression, with larger circles representing greater weight contributions. The correlation was statistically significant (p = 0.045). Heterogeneity statistics: T = 0.726, p = 0.002, I^2 = 40.85%. BMI = body mass index.

Publication	Subgroup	Difference in means	Lower limit	Upper limit	p-Value
		Statistics for each study			
El-Ati 1995 (33)	F	-0.40	-1.96	1.16	0.615
Fakhrzadeh 2003 [M] (38)	F	-0.40	-3.06	2.26	0.768
Kassab 2003 [N-Ob] (12)	F	-0.80	-4.35	2.75	0.659
Kassab 2003 [Ob] (12)	F	-0.40	-6.91	6.11	0.904
Kassab 2004 (39)	F	-0.70	-3.08	1.68	0.565
Yucel 2004 [F] (41)	F	-1.49	-4.51	1.53	0.334
Ziaee 2006 [F] (23)	F	-1.10	-2.23	0.03	0.057
Al-Hourani 2007 (46)	F	-0.60	-2.03	0.83	0.410
Moosavi 2007 [F] (48)	F	-1.10	-2.79	0.59	0.203
Lamri-Senhadji 2009 [F] (20)	F	-1.00	-11.70	9.70	0.855
Khattak 2012 [F, Nw] (14)	F	-7.40	-11.03	-3.77	0.000
Khattak 2012 [F, Ow/Ob] (14)	F	-15.40	-20.97	-9.83	0.000
Shehab 2012 [F] (57)	F	-0.60	-5.04	3.84	0.791
Norouzy 2013 [F, 18-35] (62)	F	-0.80	-2.91	1.31	0.457
Norouzy 2013 [F, 36-70] (62)	F	-0.50	-2.92	1.92	0.686
Lopez-Bueno 2015 (69)	F	-1.10	-3.40	1.20	0.348
Ongsara 2017 [M] (80)	F	-0.46	-2.25	1.33	0.615
		-1.37	-2.30	-0.45	0.004
Azizi 1992 (30)	M	-3.80	-7.54	-0.06	0.046
Adlouni 1998 (34)	M	-1.78	-4.14	0.58	0.140
Kayikcioglu 1998 (37)	M	-0.90	-2.47	0.67	0.261
Fakhrzadeh 2003 [M] (38)	M	-1.20	-2.63	0.23	0.101
Rahman 2004 (40)	M	-1.98	-4.18	0.22	0.078
Yucel 2004 [M] (41)	M	-0.16	-4.21	3.89	0.938
Al-Numair 2006 (43)	M	-2.30	-2.99	-1.61	0.000
Dewanti 2006 (22)	M	-1.50	-3.73	0.73	0.188
Subhan 2006 (45)	M	-0.52	-3.25	2.21	0.709
Ziaee 2006 [M] (23)	M	-1.20	-3.51	1.11	0.308
Mansi 2007 (47)	M	-3.98	-5.77	-2.19	0.000
Moosavi 2007 [M] (48)	M	-0.90	-2.36	0.56	0.228
Souissi 2007 (49)	M	0.43	-1.99	2.85	0.727
Houari 2008 (19)	M	-0.40	-0.83	0.03	0.071
Stannard 2008 (51)	M	-1.27	-4.39	1.85	0.424
Lamri-Senhadji 2009 [M] (20)	M	0.00	-11.92	11.92	1.000
Pathan 2010 (52)	M	-1.34	-3.85	1.17	0.296
Assadi 2011 (53)	M	-1.37	-3.40	0.66	0.185
Trabelski 2011 (24)	M	-1.50	-2.68	-0.32	0.012
Ünalacak 2011 [Ob] (13)	M	-2.90	-6.11	0.31	0.076
Ünalacak 2011 [Nw] (13)	M	-0.80	-3.13	1.53	0.502
Khattak 2012 [M, Nw] (14)	M	-6.70	-9.67	-3.73	0.000
Khattak 2012 [M, Ow/Ob] (14)	M	-15.80	-21.87	-9.73	0.000
Racinais 2012 (58)	M	-0.90	-4.73	2.93	0.645
Shehab 2012 [M] (57)	M	-1.10	-3.89	1.69	0.440
Develioglu 2013 (60)	M	-1.20	-3.62	1.22	0.331
Haouari-Oukerro 2013 (61)	M	-1.30	-1.43	-1.17	0.000
Norouzy 2013 [M, 18-35] (62)	M	-1.70	-3.81	0.41	0.114
Norouzy 2013 [M, 36-70] (62)	M	-1.20	-2.44	0.04	0.058
Sayedda 2013 (63)	M	-2.18	-4.43	0.07	0.057
Celik 2014 [Nw] (16)	M	-1.30	-3.58	0.98	0.264
Celik 2014 [Ow] (16)	M	-1.30	-3.38	0.78	0.220
Celik 2014 [Ob] (16)	M	-2.80	-8.53	2.93	0.338
Feizollahzadeh 2014 (65)	M	-1.84	-6.03	2.35	0.389
Gnanou 2015 (67)	M	-1.52	-3.78	0.74	0.187
Talib 2015 (70)	M	-0.67	-3.86	2.52	0.680
Latiri 2017 (76)	M	-0.40	-1.96	1.16	0.616
Nugraha 2017 (79)	M	-1.78	-4.88	1.32	0.261
Ongsara 2017 [F] (80)	M	-0.90	-5.05	3.25	0.671
		-1.49	-1.87	-1.10	0.000
		-1.47	-1.82	-1.12	0.000

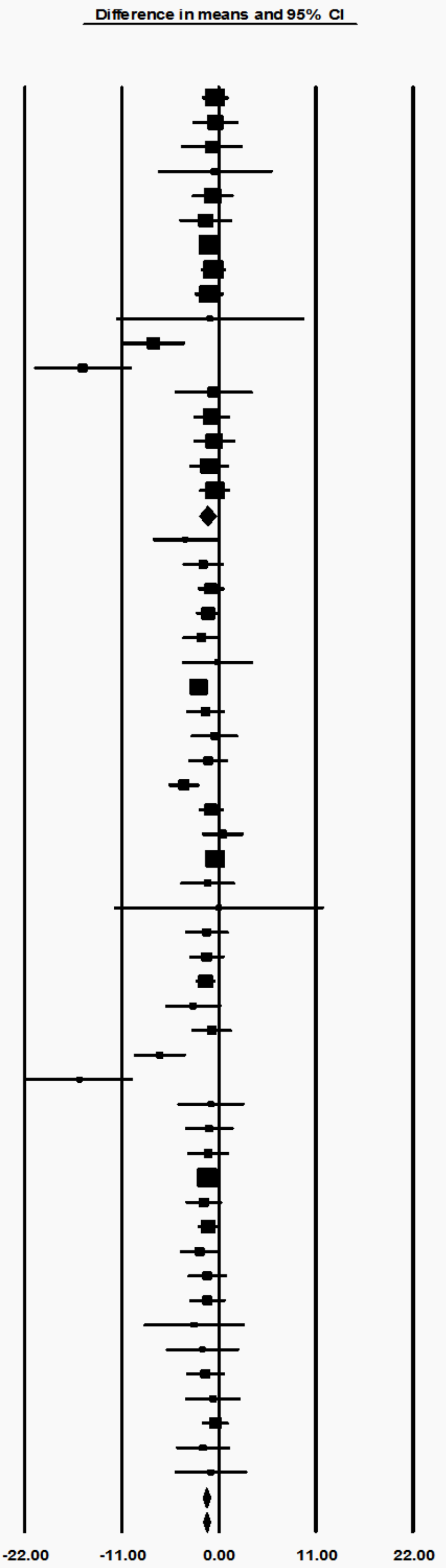

Figure S6. Weight was significantly decreased by Ramadan fasting in both the female and the male subgroup. Change in weight (kg) as subdivided by sex (female and male), between pre-Ramadan (pre-R) and end of Ramadan (post-R). There was no significant difference between female and male subgroups (p = 0.829). Heterogeneity statistics: female T = 1.376, p = 0.001, I^2 = 59.5%; male T = 0.628, p = < 0.001, I^2 = 50.8%. F = female; M = male; Nw = normal weight; Ow = overweight; Ob = obese; N-Ob = non-obese; 18-35, 36-70 = age range in years.

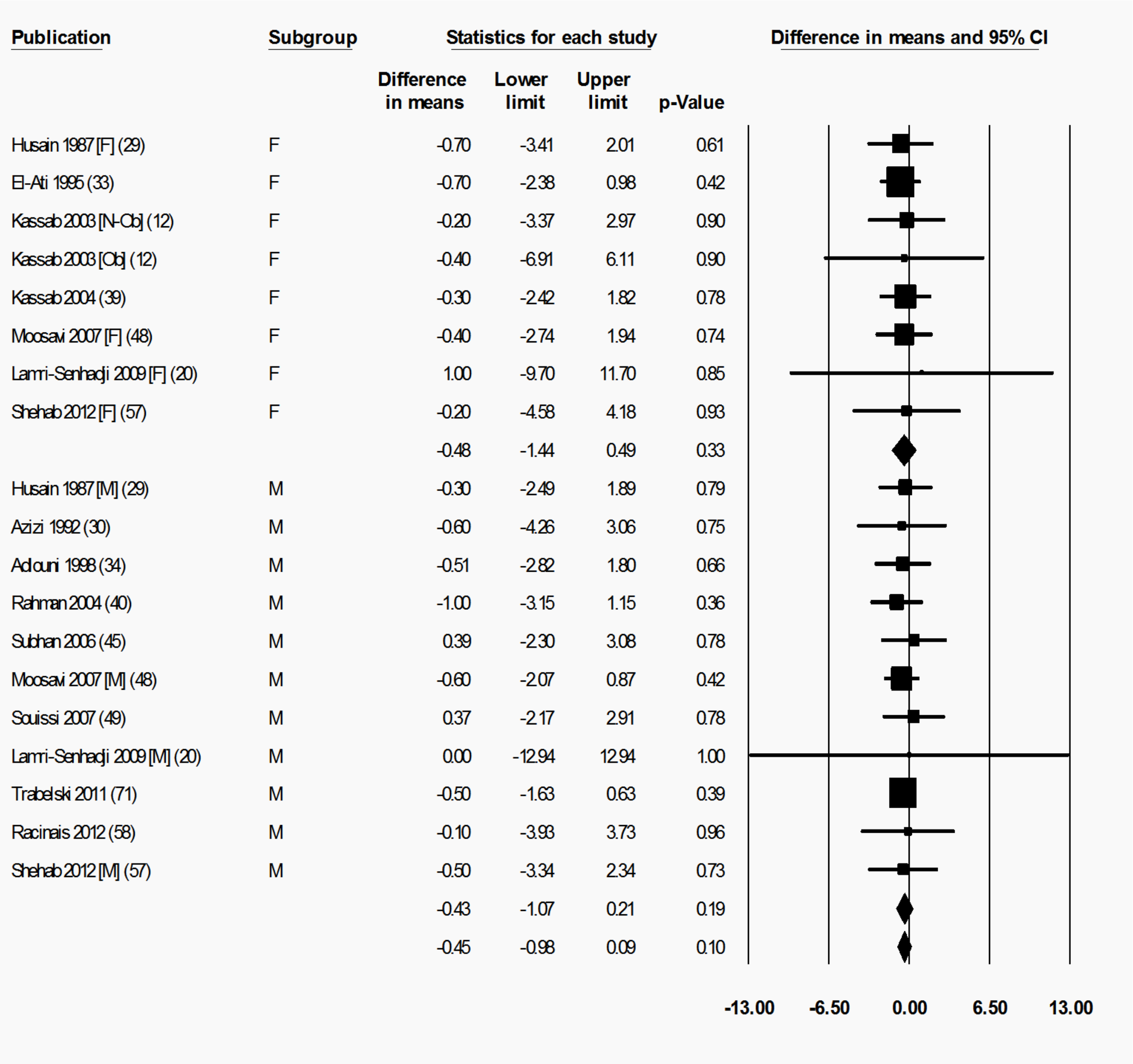

Figure S7. Weight was not significantly reduced at 2-5 weeks after Ramadan in the female or the male subgroup. Change in weight (kg) as subdivided by sex (female and male), between pre-Ramadan (pre-R) and 2-5 weeks after Ramadan (follow-up). There was no significant difference between female and male subgroups (p = 0.940). Heterogeneity statistics: female T = 0.000, p = 1.000, I^2 = 0.0%; male T = 0.000, p = 1.000, I^2 = 0.0%. F = female; M = male; Ob = obese; N-Ob = non-obese.

Publication	Subgroup	Difference in means	Lower limit	Upper limit	p-Value
Takruri 1989 [Ow] (11)	ME+NA	-2.62	-4.37	-0.87	0.003
Takruri 1989 [Nw] (11)	ME+NA	-1.99	-3.54	-0.44	0.012
Azizi 1992 (30)	ME+NA	-3.80	-7.54	-0.06	0.046
El-Ati 1995 (33)	ME+NA	-0.40	-1.96	1.16	0.615
Adlouni 1998 (34)	ME+NA	-1.78	-4.14	0.58	0.140
Bito 1998 (35)	ME+NA	-1.20	-3.62	1.22	0.332
Kayikcioglu 1998 (37)	ME+NA	-0.90	-2.47	0.67	0.261
Fakhrzadeh 2003 [M] (38)	ME+NA	-1.20	-2.63	0.23	0.101
Fakhrzadeh 2003 [F] (38)	ME+NA	-0.40	-3.06	2.26	0.768
Kassab 2003 [N-Ob] (12)	ME+NA	-0.80	-4.35	2.75	0.659
Kassab 2003 [Ob] (12)	ME+NA	-0.40	-6.91	6.11	0.904
Kassab 2004 (39)	ME+NA	-0.70	-3.08	1.68	0.565
Saleh 2004 (18)	ME+NA	-1.50	-3.89	0.89	0.219
Yucel 2004 [M] (41)	ME+NA	-0.16	-4.21	3.89	0.938
Yucel 2004 [F] (41)	ME+NA	-1.49	-4.51	1.53	0.334
Aksungar 2005 (42)	ME+NA	0.23	-1.24	1.70	0.760
Al-Numair 2006 (43)	ME+NA	-2.30	-2.99	-1.61	0.000
Lamine 2006 (44)	ME+NA	0.40	-1.93	2.73	0.737
Ziaee 2006 [M] (23)	ME+NA	-1.20	-3.51	1.11	0.308
Ziaee 2006 [F] (23)	ME+NA	-1.10	-2.23	0.03	0.057
Al-Hourani 2007 (46)	ME+NA	-0.60	-2.03	0.83	0.410
Mansi 2007 (47)	ME+NA	-3.98	-5.37	-2.59	0.000
Moosavi 2007 [M] (48)	ME+NA	-0.90	-2.36	0.56	0.228
Moosavi 2007 [F] (48)	ME+NA	-1.10	-2.79	0.59	0.202
Haouari 2008 (19)	ME+NA	-0.40	-0.83	0.03	0.071
Ibrahim 2008 (50)	ME+NA	-1.40	-4.96	2.16	0.441
Lamri-Senhadji 2009 [M] (20)	ME+NA	-1.00	-11.70	9.70	0.855
Lamri-Senhadji 2009 [F] (20)	ME+NA	0.00	-11.92	11.92	1.000
Assadi 2011 (53)	ME+NA	-1.37	-3.40	0.66	0.185
Trabelsi 2011 (24)	ME+NA	-1.50	-2.68	-0.32	0.012
Ünalacak 2011 [Ob] (13)	ME+NA	-2.90	-6.11	0.31	0.076
Ünalacak 2011 [Nw] (13)	ME+NA	-0.80	-3.13	1.53	0.502
Faris 2012a (54)	ME+NA	-1.24	-3.57	1.09	0.298
Faris 2012b (55)	ME+NA	-0.80	-3.23	1.63	0.519
Racinais 2012 (58)	ME+NA	-0.90	-4.73	2.93	0.645
Shehab 2012 [M] (57)	ME+NA	-1.10	-3.89	1.69	0.440
Shehab 2012 [F] (57)	ME+NA	-0.60	-5.04	3.84	0.791
Develioglu 2013 (60)	ME+NA	-1.20	-3.62	1.22	0.331
Haouari-Oukerro 2013 (61)	ME+NA	-1.30	-1.43	-1.17	0.000
Norouzy 2013 [M, 18-35] (62)	ME+NA	-1.70	-3.81	0.41	0.114
Norouzy 2013 [F, 18-35] (62)	ME+NA	-0.80	-2.91	1.31	0.457
Norouzy 2013 [M, 36-70] (62)	ME+NA	-1.20	-2.44	0.04	0.058
Norouzy 2013 [F, 36-70] (62)	ME+NA	-0.50	-2.92	1.92	0.686
Akaberi 2014 (64)	ME+NA	-1.40	-3.64	0.84	0.220
Cansel 2014 (66)	ME+NA	0.50	-8.58	9.58	0.914
Celik 2014 [Nw] (16)	ME+NA	-1.30	-3.58	0.98	0.264
Celik 2014 [Ow] (16)	ME+NA	-1.30	-3.38	0.78	0.220
Celik 2014 [Ob] (16)	ME+NA	-2.80	-8.53	2.93	0.338
Talib 2015 (70)	ME+NA	-0.67	-3.86	2.52	0.680
Latiri 2017 (76)	ME+NA	-0.80	-4.36	2.76	0.660
Malekmakan 2017 (77)	ME+NA	-1.20	-3.36	0.96	0.277
Norouzy 2017 (78)	ME+NA	0.10	-5.97	6.17	0.974
Almeneessier 2017 (81)	ME+NA	-2.80	-8.01	2.41	0.292
Almeneessier 2018 (82)	ME+NA	-1.60	-6.12	2.92	0.488
		-1.22	-1.43	-1.01	0.000
Rahman 2004 (40)	SA	-1.98	-4.18	0.22	0.078
Subhan 2006 (45)	SA	-0.52	-3.25	2.21	0.709
Pathan 2010 (52)	SA	-1.34	-3.85	1.17	0.296
Sayedda 2013 (63)	SA	-2.18	-4.43	0.07	0.057
Khan 2017 (74)	SA	-0.03	-4.38	4.32	0.989
Kiyani 2017 (75)	SA	-0.40	-1.96	1.16	0.616
		-1.11	-2.04	-0.18	0.019
Dewanti 2006 (22)	SEA	-1.50	-3.73	0.73	0.188
Khattak 2012 [M, Nw] (14)	SEA	-6.70	-9.67	-3.73	0.000
Khattak 2012 [M, Ob] (14)	SEA	-15.80	-21.87	-9.73	0.000
Khattak 2012 [F, Nw] (14)	SEA	-7.40	-11.03	-3.77	0.000
Khattak 2012 [F, Ob] (14)	SEA	-15.40	-20.97	-9.83	0.000
Rohin 2013 [Nw] (15)	SEA	-1.27	-3.46	0.92	0.256
Rohin 2013 [Ow] (15)	SEA	-1.29	-3.38	0.80	0.227
Rohin 2013 [Ob] (15)	SEA	-1.66	-5.65	2.33	0.415
Gnanou 2015 (67)	SEA	-1.52	-3.78	0.74	0.187
Syam 2016 (26)	SEA	0.03	-2.09	2.15	0.978
Ongsara 2017 [M] (80)	SEA	-0.90	-5.05	3.25	0.671
Ongsara 2017 [F] (80)	SEA	-0.46	-2.25	1.33	0.615
		-3.68	-5.67	-1.68	0.000
Sweileh 1992 (31)	WC	-1.92	-7.17	3.33	0.473
Finch 1998 (36)	WC	-0.30	-2.66	2.06	0.803
Stannard 2008 (51)	WC	-1.27	-4.39	1.85	0.424
Hajek 2012 (56)	WC	-0.63	-2.28	1.03	0.458
McNeil 2014 [Nw] (17)	WC	-1.00	-4.65	2.65	0.591
McNeil 2014 [Ob] (17)	WC	-2.60	-8.70	3.50	0.403
Nugraha 2017 (79)	WC	-1.78	-4.88	1.32	0.261
		-0.91	-1.97	0.15	0.091
		-1.23	-1.43	-1.03	0.000

Statistics for each study

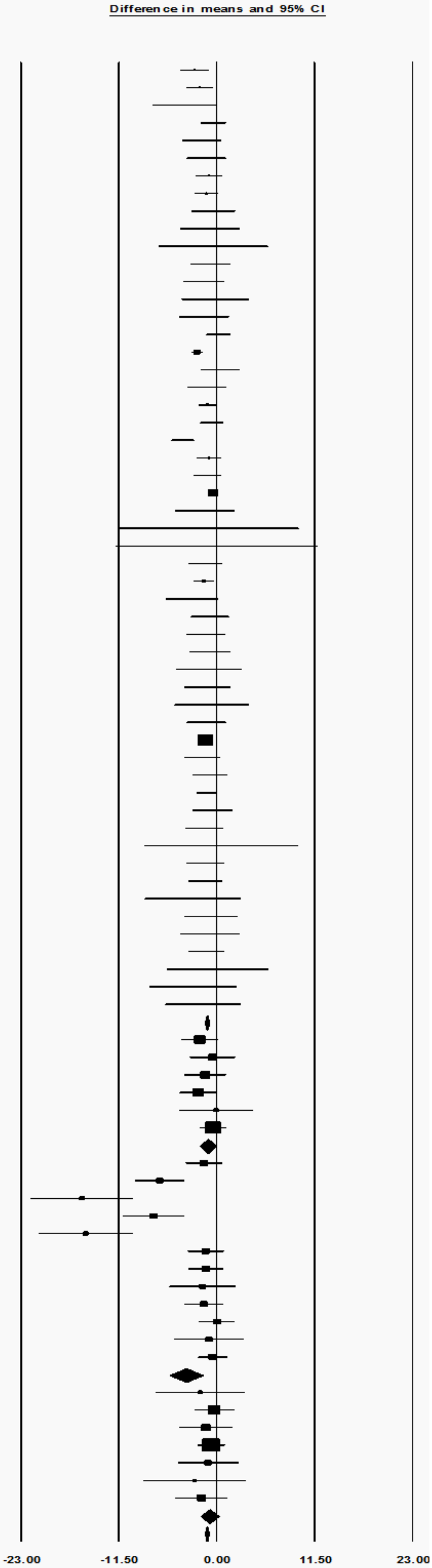

Figure S8. Weight was significantly decreased by Ramadan fasting in the the Middle East + North Africa, South Asia, and South East Asia, but not in the Westernized countries subgroup. Change in weight (kg) as subdivided by location category (Middle East + North Africa, South Asia, South East Asia, and Westernized countries), between pre-Ramadan (pre-R) and the end of Ramadan (post-R). There was no significant difference between subgroups ($p = 0.069$). Heterogeneity statistics: Middle East + North Africa $T = 0.185$, $p = 0.322$, $I^2 = 7.4\%$; South Asia $T = 0.000$, $p = 0.744$, $I^2 = 0.0\%$; South East Asia $T = 9.667$, $p = < 0.001$, $I^2 = 83.8\%$; Westernized countries $T = 0.000$, $p = 0.979$, $I^2 = 0.0\%$. F = female; M = male; Nw = normal weight; Ow = overweight; Ob = obese; N-Ob = non-obese; 18-35, 36-70 = age range in years; ME + NA = Middle East + North Africa; SA = South Asia; SEA = South East Asia; WC = westernized countries.

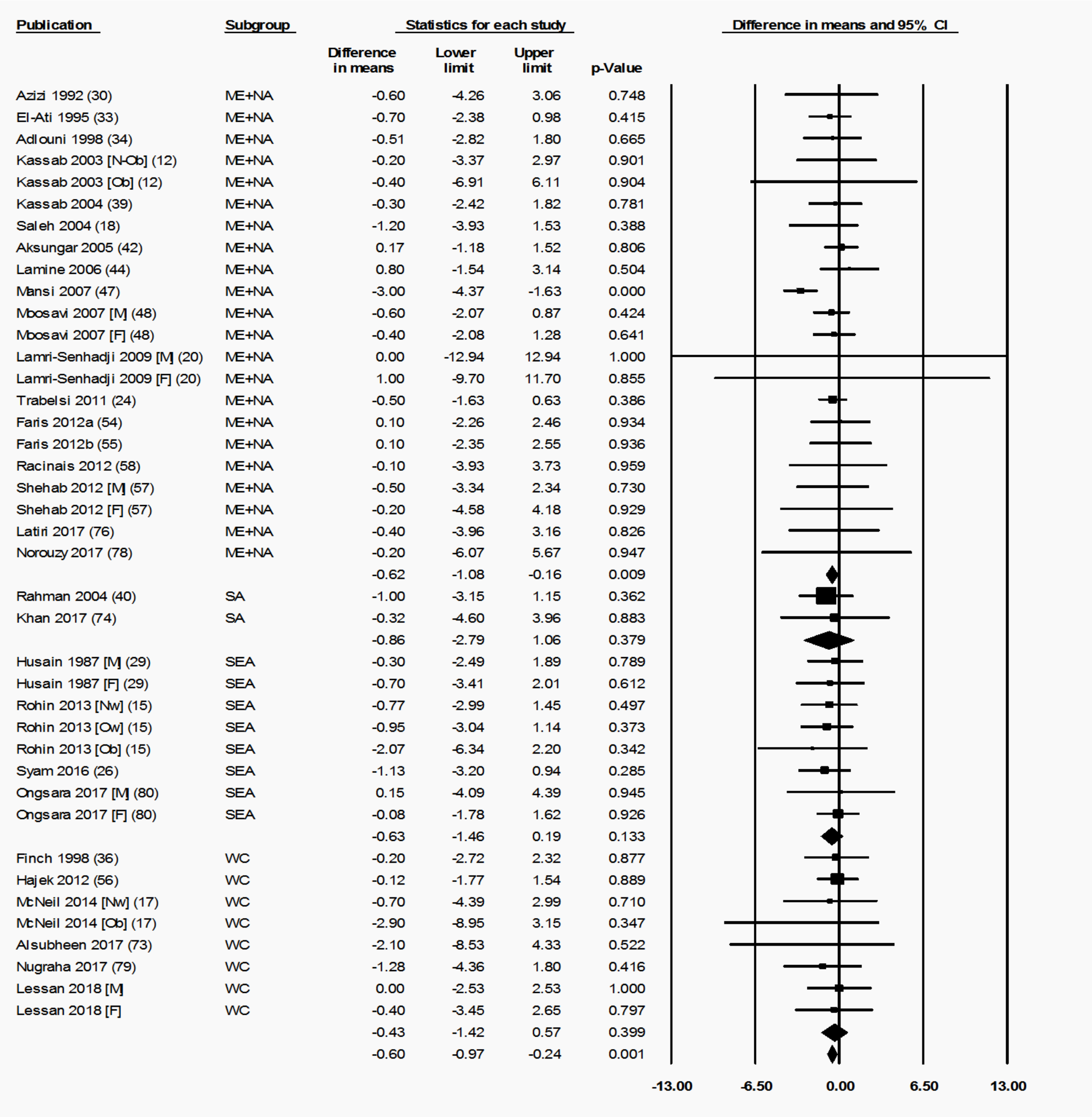

Figure S9. Weight was not significantly reduced at 2-5 weeks after Ramadan only in the Middle East + North Africa but not in any other subgroup. Change in weight (kg) as subdivided by location category (Middle East + North Africa, South Asia, South East Asia and Westernized countries), between pre-Ramadan (pre-R) and 2-5 weeks after Ramadan (follow-up). Heterogeneity statistics: Middle East + North Africa $T = 0.000$, $p = 0.785$, $I^2 = 0.0\%$; South Asia $T = 0.000$, $p = 0.781$, $I^2 = 0.0\%$; South East Asia $T = 0.000$, $p = 0.986$, $I^2 = 0.0\%$; Westernized countries $T = 0.000$, $p = 0.983$, $I^2 = 0.0\%$. F = female; M = male; Nw = normal weight; Ow = overweight; Ob = obese; N-Ob = non-obese; ME + NA = Middle East + North Africa; SA = South Asia; SEA = South East Asia; WC = westernized countries.

(a)

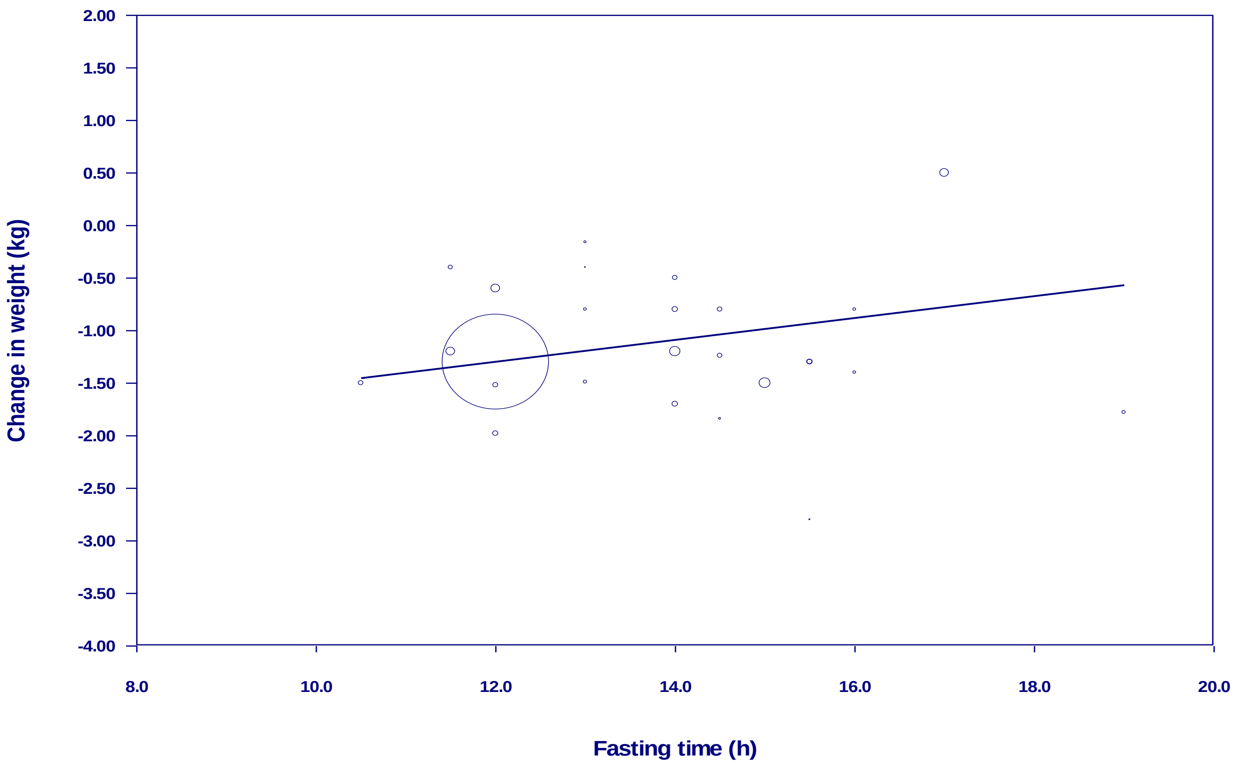
2.00
1.50
1.00
0.50
0.00
-0.50
-1.00
-1.50
-2.00
-2.50
-3.00
-3.50
-4.00
Change in weight (kg)
8.0
10.0
12.0
14.0
16.0
18.0
20.0
Fasting time (h)

(b)

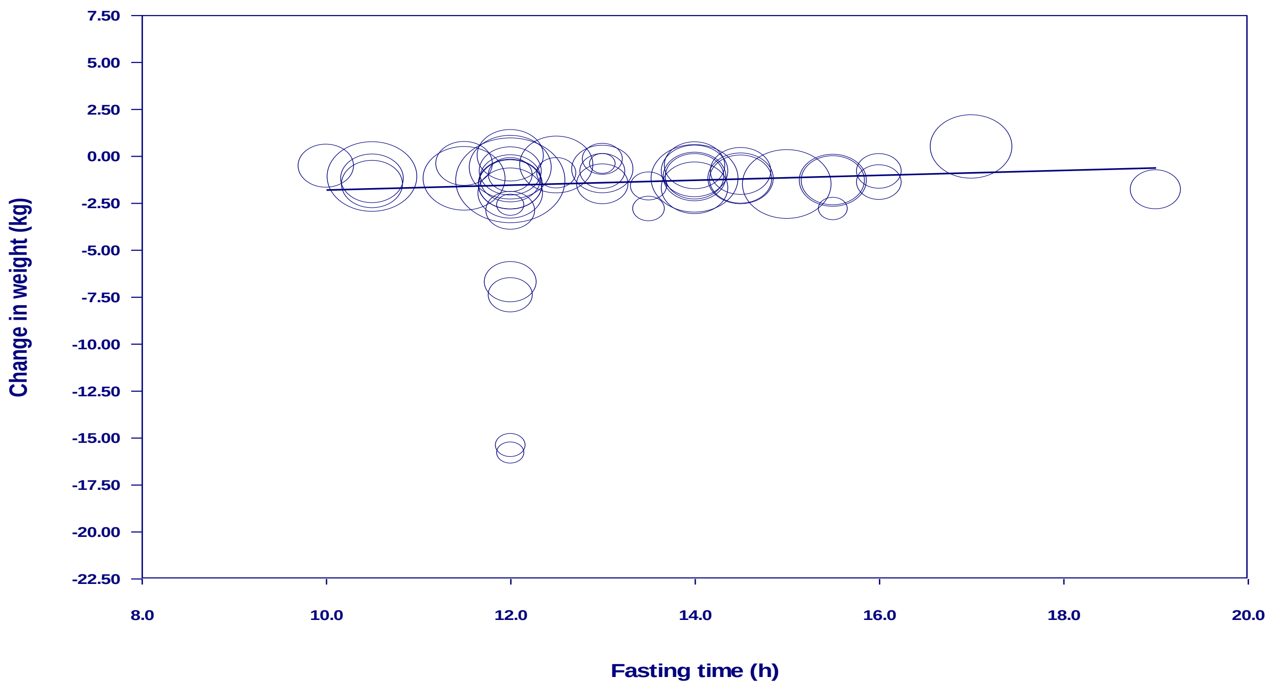
7.50
5.00
2.50
0.00
-2.50
-5.00
-7.50
-10.00
-12.50
-15.00
-17.50
-20.00
-22.50
Change in weight (kg)
8.0
10.0
12.0
14.0
16.0
18.0
20.0
Fasting time (h)

Figure S10. There was no significant correlation between fasting time and mean weight change, either when using only published mean fasting times (a), or when using both published and inferred mean fasting times (b). Scatterplot of the meta-regression analysis for the correlation between fasting time and weight change from pre-Ramadan (pre-R) to the end of Ramadan (post-R) for each of the 42 (a) or 75 (b) comparison groups from publications that obtained both fasting time and weight data at these two time points (pre-R and post-R). The area of each circle represents the percentage weight contributed by each study to the overall meta-regression, with larger circles representing greater weight contributions. The correlation was not statistically significant for either meta-regression (p = 0.226 and p = 0.794 for (a) and (b), respectively). Heterogeneity statistics: T = 0.143, p = 0.395, I^2 = 4.2% and T = 0.693, p < 0.001, I^2 = 46.5% for (a) and (b) respectively.

Publication	Statistics for each study			
	Difference in means	Lower limit	Upper limit	p-Value
Kassab 2003 [N-Ob] (12)	-0.10	-2.12	1.92	0.923
Kassab 2003 [Ob] (12)	0.10	-1.26	1.46	0.886
Kassab 2004 (39)	0.10	-1.90	2.10	0.922
Saleh 2004 (18)	-3.82	-5.08	-2.56	0.000
Trabelsi 2011 (24)	-0.50	-1.06	0.06	0.081
Faris 2012a (54)	6.36	4.24	8.48	0.000
Faris 2012b (55)	6.00	3.98	8.02	0.000
Agoumi 2013 (59)	0.92	-0.98	2.82	0.342
Rohin 2013 [Nw] (15)	-1.02	-2.69	0.65	0.232
Rohin 2013 [Ow] (15)	-0.52	-1.89	0.85	0.458
Rohin 2013 [Ob] (15)	-0.72	-2.55	1.11	0.441
Ongsara 2017 [M] (80)	2.89	0.60	5.18	0.013
Ongsara 2017 [F] (80)	-0.21	-1.29	0.87	0.704
	0.61	-0.62	1.83	0.330

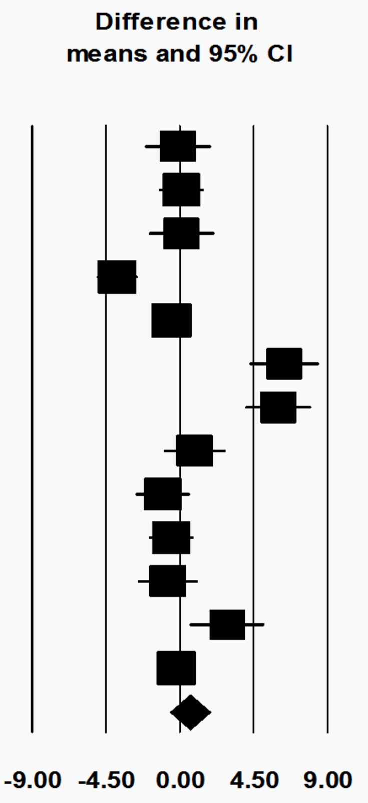

Figure S11. Fat percentage was not significantly reduced at 2-5 weeks after Ramadan fasting. Change in fat percentage (%) between pre-Ramadan (pre-R) and 2-5 weeks after Ramadan (follow-up). Heterogeneity statistics: $T = 2.083$, $p < 0.001$, $I^2 = 89.7\%$. F = female; M = male; Nw = normal weight; Ow = overweight; Ob = obese; N-Ob = non-obese.

Publication	Subgroup	Statistics for each study			
		Difference in means	Lower limit	Upper limit	p-Value
Kassab 2003 [N-Ob] (12)	Nw	-0.10	-2.13	1.93	0.923
Rohin 2013 [Nw] (15)	Nw	-1.02	-2.09	0.05	0.062
		-0.82	-1.77	0.13	0.091
Kassab 2003 [Ob] (12)	Ow/Ob	0.10	-1.27	1.47	0.887
Saleh 2004 (18)	Ow/Ob	-3.82	-5.08	-2.56	0.000
Rohin 2013 [Ow] (15)	Ow/Ob	-0.52	-1.39	0.35	0.239
Rohin 2013 [Ob] (15)	Ow/Ob	-0.72	-1.53	0.09	0.080
		-1.22	-2.66	0.23	0.099
		-0.94	-1.73	-0.15	0.020

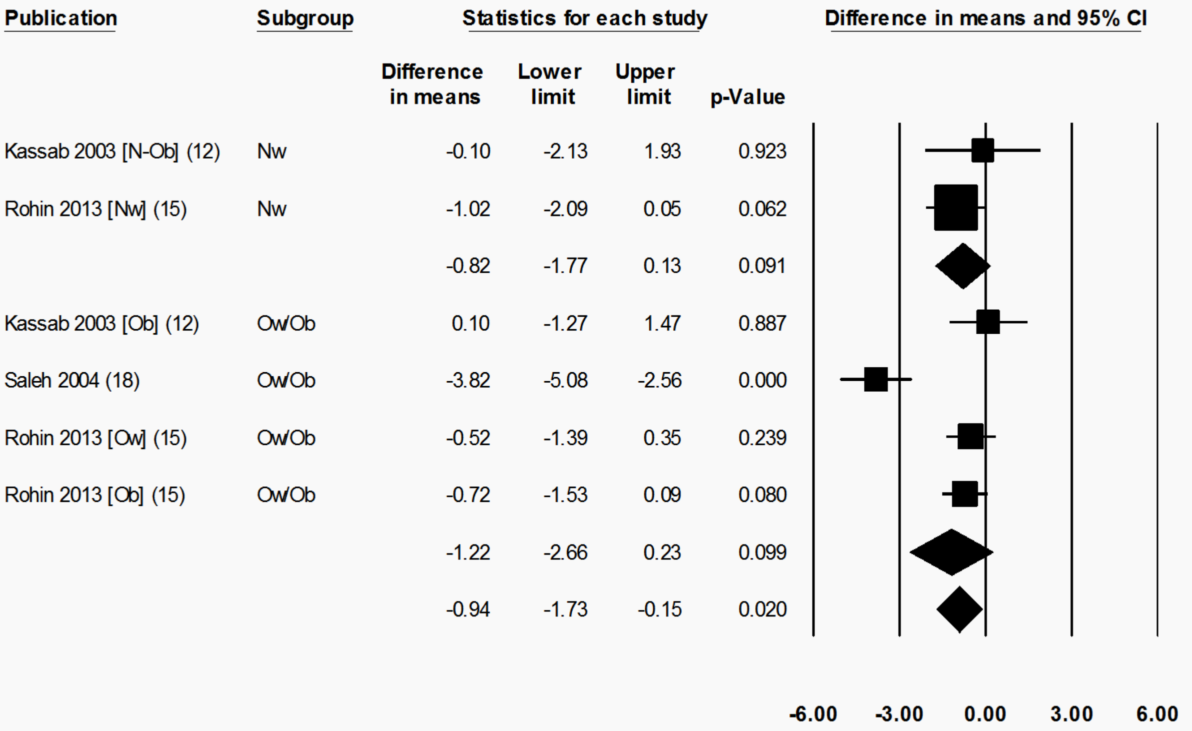

Figure S12. Fat percentage was not significantly reduced at 2-5 weeks after Ramadan fasting in the normal weight or the overweight/obese subgroup. Change in fat percentage as subdivided by BMI category (normal weight and overweight/obese), between pre-Ramadan (pre-R) and 2-5 weeks after Ramadan (follow-up). There was no significant difference between normal weight and overweight/obese subgroups (p = 0.651). Heterogeneity statistics: normal weight T = 0.000, p = 0.432, I^2 = 0.0%; overweight/obese T = 1.365, p < 0.001, I^2 = 87.1%. Nw = normal weight; Ow = overweight; Ob = obese.

Publication	Subgroup	Statistics for each study			
		Difference in means	Lower limit	Upper limit	p-Value
Kassab 2003 [N-Ob] (12)	F	-0.10	-2.13	1.93	0.923
Kassab 2003 [Ob] (12)	F	0.10	-1.27	1.47	0.887
Kassab 2004 (39)	F	0.10	-1.90	2.10	0.922
Ongsara 2017 [F] (80)	F	-0.21	-1.29	0.87	0.704
		-0.07	-0.80	0.66	0.858
Trabelski 2011 (24)	M	-0.50	-1.06	0.06	0.081
Ongsara 2017 [M] (80)	M	2.89	0.60	5.18	0.013
		1.01	-2.30	4.31	0.550
		-0.02	-0.73	0.70	0.964

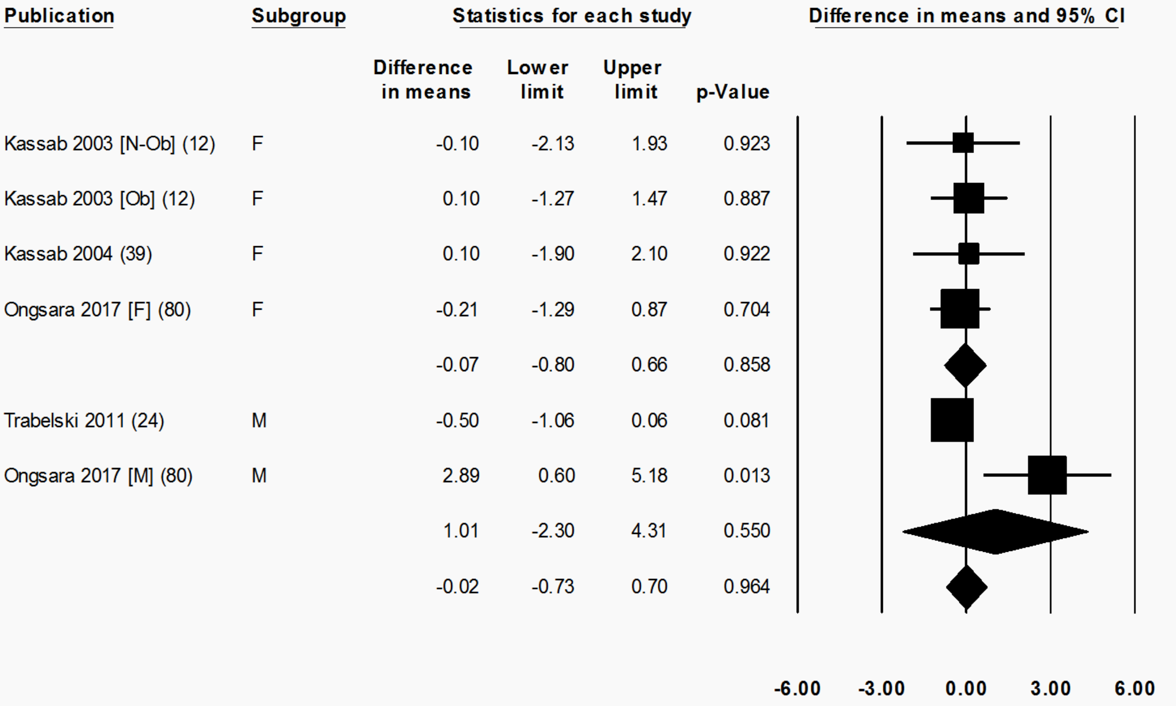

Figure S13. Fat percentage was not significantly reduced at 2-5 weeks after Ramadan fasting in the female or the male subgroup. Change in fat percentage as subdivided by sex (female and male), between pre-Ramadan (pre-R) and 2-5 weeks after Ramadan (follow-up). There was no significant difference between female and male subgroups (p = 0.534). Heterogeneity statistics: female T = 0.000, p = 0.985, I^2 = 0.0%; male T = 2.242, p = 0.005, I^2 = 87.5%. F = female; M = male; Ob = obese; N-Ob = non-obese.

Publication	Statistics for each study			
	Difference in means	Lower limit	Upper limit	p-Value
El-Ati 1995 (33)	-0.60	-2.16	0.96	0.451
Kassab 2003 [N-Ob] (12)	-0.10	-1.75	1.55	0.906
Kassab 2003 [Ob] (12)	-0.10	-3.14	2.94	0.949
Kassab 2004 (39)	-0.20	-2.50	2.10	0.865
Racinais 2012 (58)	0.50	-1.75	2.75	0.663
McNeil 2014 [Nw] (17)	-0.20	-2.03	1.63	0.830
McNeil 2014 [Ob] (17)	-1.30	-5.24	2.64	0.517
Ongsara 2017 [M] (80)	2.18	-0.50	4.86	0.110
Ongsara 2017 [F] (80)	-0.20	-1.24	0.84	0.707
	-0.09	-0.70	0.51	0.760

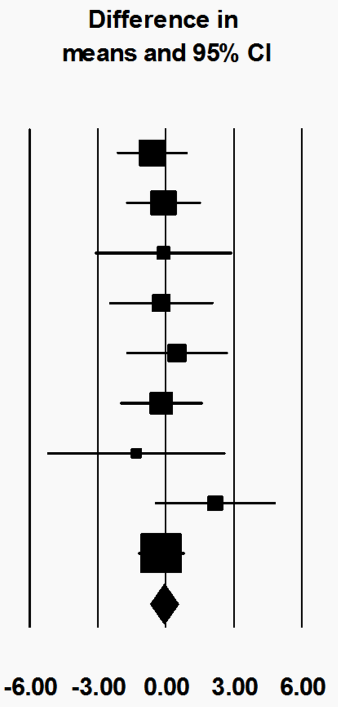

Figure S14. Absolute fat mass was not significantly reduced at 2-5 weeks after Ramadan fasting. Change in absolute fat mass (kg) between pre-Ramadan (pre-R) and 2-5 weeks after Ramadan (follow-up). Heterogeneity statistics: $T = 0.00$, $p = 0.869$, $I^2 = 0.0\%$. F = female; M = male; Nw = normal weight; Ob = obese; N-Ob = non-obese.

Publication	Subgroup	Difference in means	Lower limit	Upper limit	p-Value
El-Ati 1995 (33)	F	-0.60	-2.16	0.96	0.451
Kassab 2003 [N-Ob] (12)	F	-0.10	-1.75	1.55	0.905
Kassab 2003 [Ob] (12)	F	-0.10	-3.14	2.94	0.949
Kassab 2004 (39)	F	-0.20	-2.50	2.10	0.865
Ongsara 2017 [F] (80)	F	-0.20	-1.24	0.84	0.707
		-0.26	-0.97	0.45	0.474
Racinais 2012 (58)	M	0.50	-1.75	2.75	0.663
Ongsara 2017 [M] (80)	M	2.18	-0.50	4.86	0.110
		1.20	-0.53	2.92	0.173
		-0.05	-0.70	0.61	0.885

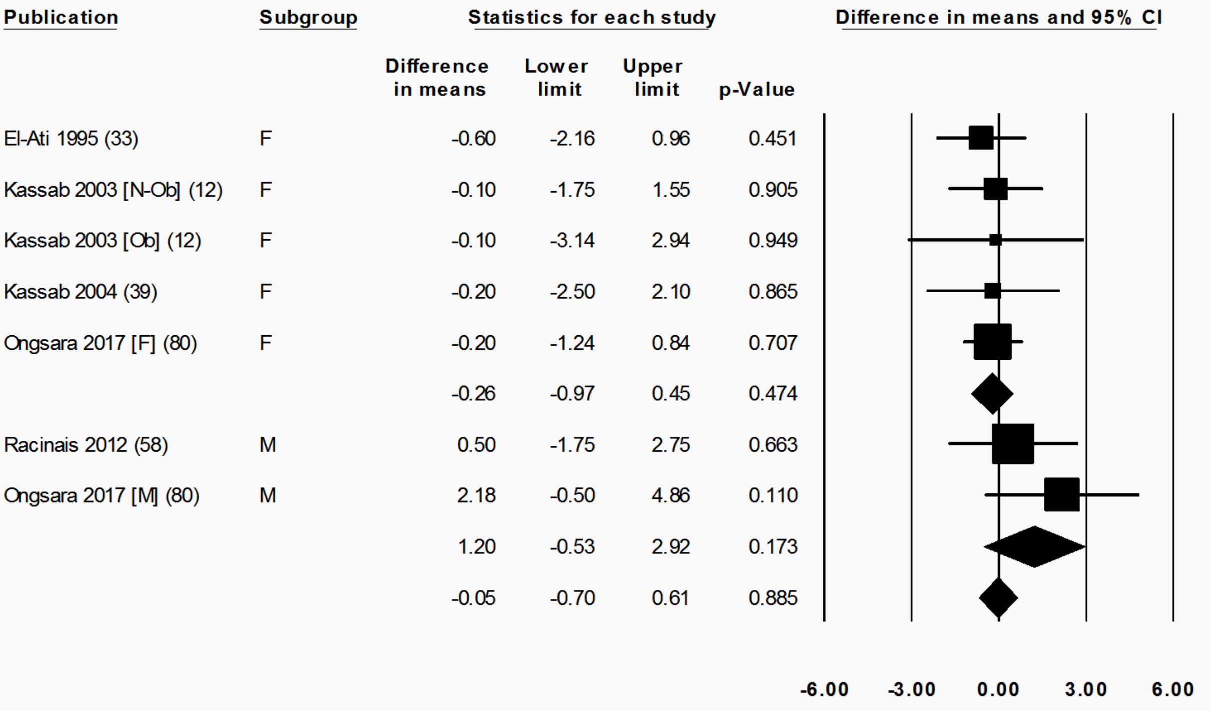

Figure S15. Absolute fat mass was not significantly reduced at 2-5 weeks after Ramadan fasting in the female or the male subgroup. Change in absolute fat mass (kg) as subdivided by sex (female and male), between pre-Ramadan (pre-R) and 2-5 weeks after Ramadan (follow-up). There was no significant difference between female and male subgroups (p = 0.126). Heterogeneity statistics: female T = 0.000, p = 0.993, I^2 = 0.0%; male T = 0.000, p = 0.346, I^2 = 0.0%. F = female; M = male; Ob = obese; N-Ob = non-obese.

Publication	Statistics for each study			
	Difference in means	Lower limit	Upper limit	p-Value
El-Ati 1995 (33)	-0.20	-1.03	0.63	0.635
Trabelsi 2011 (24)	0.10	-1.10	1.30	0.870
Racinais 2012 (58)	-0.20	-2.65	2.25	0.873
McNeil 2014 [Nw] (17)	-0.50	-2.78	1.78	0.667
McNeil 2014 [Ob] (17)	-1.20	-4.76	2.36	0.509
Ongsara 2017 [M] (80)	-1.99	-4.21	0.23	0.079
Ongsara 2017 [F] (80)	-0.10	-0.86	0.66	0.796
	-0.23	-0.69	0.24	0.346

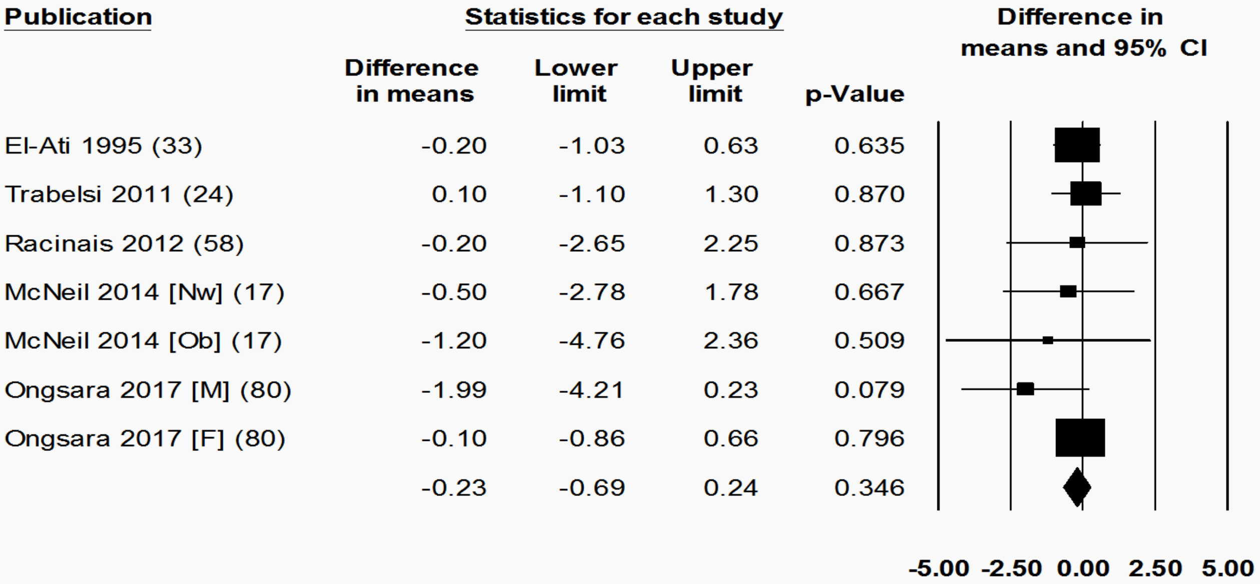

Figure S16. Fat-free mass was not significantly reduced at 2-5 weeks after Ramadan fasting. Change in fat-free mass (kg) between pre-Ramadan (pre-R) and 2-5 weeks after Ramadan (follow-up). Heterogeneity statistics: $T = 0.000$, $p = 0.788$, $I^2 = 0.0\%$. F = female; M = male; Nw = normal weight; Ob = obese.

Publication	Subgroup	Difference in means	Lower limit	Upper limit	p-Value
El-Ati 1995 (33)	F	-0.20	-1.03	0.63	0.635
Ongsara 2017 [F] (80)	F	-0.10	-0.86	0.66	0.796
		-0.15	-0.70	0.41	0.609
Trabelski 2011 (24)	M	0.10	-1.10	1.30	0.870
Racinais 2012 (57)	M	-0.20	-2.65	2.25	0.873
Ongsara 2017 [M] (80)	M	-1.99	-4.21	0.23	0.079
		-0.46	-1.67	0.75	0.455
		-0.20	-0.71	0.31	0.437

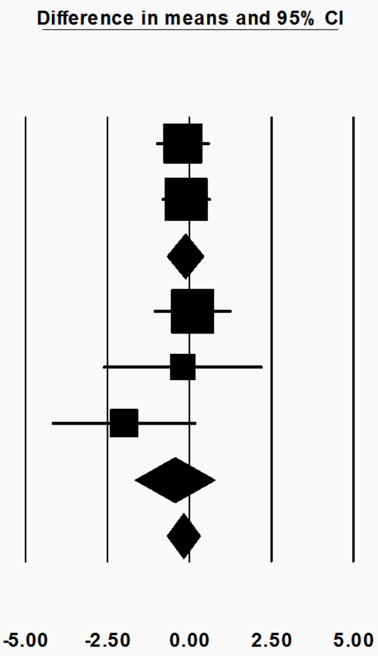

Figure S17. Fat-free mass was not significantly reduced at 2-5 weeks after Ramadan fasting in the female or the male subgroup. Change in fat-free mass (kg) as subdivided by sex (female and male), between pre-Ramadan (pre-R) and 2-5 weeks after Ramadan (follow-up). There was no significant difference between female and male subgroups (p = 0.126). Heterogeneity statistics: female T = 0.000, p = 0.861, I^2 = 0.0%; male T = 0.265, p = 0.265, I^2 = 24.7%. F = female; M = male.

Publication	Statistics for each study			
	Hedges's g	Lower limit	Upper limit	p-Value
Sweileh 1992 (31)	0.28	0.06	0.51	0.012
Souissi 2007 (49)	-0.16	-0.33	0.02	0.079
Racinais 2012 (58)	-0.01	-0.17	0.15	0.909
	0.03	-0.20	0.26	0.797

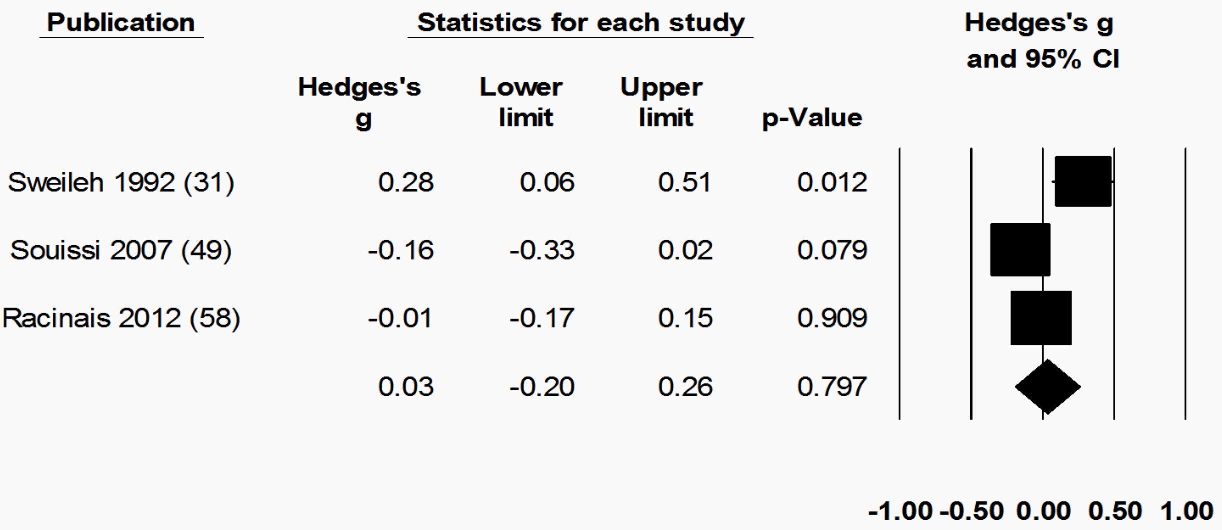

Figure S18. Maximum effort physical activity was not significantly changed by Ramadan fasting. Change in maximum effort physical activity (Hedges' g or corrected standardised mean difference) between pre-Ramadan (pre-R) and the end of Ramadan (post-R). Heterogeneity statistics: $T = 0.179$, $p = 0.009$, $I^2 = 78.7\%$.

Publication	Statistics for each study			
	Hedges's g	Lower limit	Upper limit	p-Value
Alsubheen 2017 (73)	0.20	-0.18	0.57	0.309
Khan 2017 (74)	-0.15	-0.43	0.13	0.299
Nugraha 2017 (79)	-0.31	-0.56	-0.07	0.013
	-0.12	-0.39	0.15	0.381

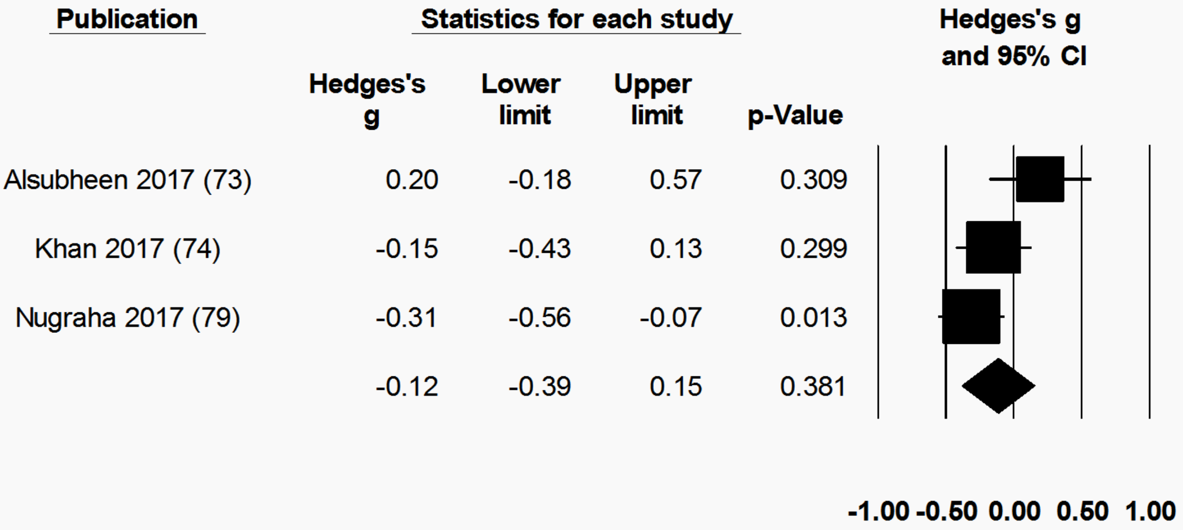

Figure S19. Daily physical activity was not significantly changed by Ramadan fasting. Change in daily physical activity (Hedges' g or corrected standardised mean difference) between pre-Ramadan (pre-R) and the end of Ramadan (post-R). Heterogeneity statistics: $T = 0.182$, $p = 0.086$, $I^2 = 59.3\%$.

Table S2. PRISMA checklist.

Section/topic	#	Checklist item	Reported on page #
**TITLE**			
Title	1	Identify the report as a systematic review, meta-analysis, or both.	1
**ABSTRACT**			
Structured summary	2	Provide a structured summary including, as applicable: background; objectives; data sources; study eligibility criteria, participants, and interventions; study appraisal and synthesis methods; results; limitations; conclusions and implications of key findings; systematic review registration number.	1
**INTRODUCTION**			
Rationale	3	Describe the rationale for the review in the context of what is already known.	2
Objectives	4	Provide an explicit statement of questions being addressed with reference to participants, interventions, comparisons, outcomes, and study design (PICOS).	2
**METHODS**			
Protocol and registration	5	Indicate if a review protocol exists, if and where it can be accessed (e.g., Web address), and, if available, provide registration information including registration number.	–
Eligibility criteria	6	Specify study characteristics (e.g., PICOS, length of follow-up) and report characteristics (e.g., years considered, language, publication status) used as criteria for eligibility, giving rationale.	2
Information sources	7	Describe all information sources (e.g., databases with dates of coverage, contact with study authors to identify additional studies) in the search and date last searched.	2-3
Search	8	Present full electronic search strategy for at least one database, including any limits used, such that it could be repeated.	2
Study selection	9	State the process for selecting studies (i.e., screening, eligibility, included in systematic review, and, if applicable, included in the meta-analysis).	2-3
Data collection process	10	Describe method of data extraction from reports (e.g., piloted forms, independently, in duplicate) and any processes for obtaining and confirming data from investigators.	3
Data items	11	List and define all variables for which data were sought (e.g., PICOS, funding sources) and any assumptions and simplifications made.	3
Risk of bias in individual studies	12	Describe methods used for assessing risk of bias of individual studies (including specification of whether this was done at the study or outcome level), and how this information is to be used in any data synthesis.	–
Summary measures	13	State the principal summary measures (e.g., risk ratio, difference in means).	4
Synthesis of results	14	Describe the methods of handling data and combining results of studies, if done, including measures of consistency (e.g., I^2) for each meta-analysis.	4-5

Section/topic	#	Checklist item	Reported on page #
Risk of bias across studies	15	Specify any assessment of risk of bias that may affect the cumulative evidence (e.g., publication bias, selective reporting within studies).	4
Additional analyses	16	Describe methods of additional analyses (e.g., sensitivity or subgroup analyses, meta-regression), if done, indicating which were pre-specified.	4
**RESULTS**			
Study selection	17	Give numbers of studies screened, assessed for eligibility, and included in the review, with reasons for exclusions at each stage, ideally with a flow diagram.	5
Study characteristics	18	For each study, present characteristics for which data were extracted (e.g., study size, PICOS, follow-up period) and provide the citations.	Supplementary 2-5
Risk of bias within studies	19	Present data on risk of bias of each study and, if available, any outcome level assessment (see item 12).	_
Results of individual studies	20	For all outcomes considered (benefits or harms), present, for each study: (a) simple summary data for each intervention group (b) effect estimates and confidence intervals, ideally with a forest plot.	8-14, Supplementary 6-37
Synthesis of results	21	Present results of each meta-analysis done, including confidence intervals and measures of consistency.	6-14, Supplementary 14-37
Risk of bias across studies	22	Present results of any assessment of risk of bias across studies (see Item 15).	_
Additional analysis	23	Give results of additional analyses, if done (e.g., sensitivity or subgroup analyses, meta-regression [see Item 16]).	Supplementary 20, 27, 28
**DISCUSSION**			
Summary of evidence	24	Summarize the main findings including the strength of evidence for each main outcome; consider their relevance to key groups (e.g., healthcare providers, users, and policy makers).	14,15,17
Limitations	25	Discuss limitations at study and outcome level (e.g., risk of bias), and at review-level (e.g., incomplete retrieval of identified research, reporting bias).	17
Conclusions	26	Provide a general interpretation of the results in the context of other evidence, and implications for future research.	15-17
**FUNDING**			
Funding	27	Describe sources of funding for the systematic review and other support (e.g., supply of data); role of funders for the systematic review.	18

From: Moher D, Liberati A, Tetzlaff J, Altman DG, The PRISMA Group (2009). Preferred Reporting Items for Systematic Reviews and Meta-Analyses: The PRISMA Statement. PLoS Med 6(7): e1000097. doi:10.1371/journal.pmed1000097 For more information, visit: **www.prisma-statement.org**.
